# Rescuing Tetracycline Class Antibiotics for the Treatment of Multidrug-Resistant Acinetobacter baumannii Pulmonary Infection

**DOI:** 10.1128/mbio.03517-21

**Published:** 2022-01-11

**Authors:** David M. P. De Oliveira, Brian M. Forde, Minh-Duy Phan, Bernhard Steiner, Bing Zhang, Johannes Zuegg, Ibrahim M. El-deeb, Gen Li, Nadia Keller, Stephan Brouwer, Nichaela Harbison-Price, Amanda J. Cork, Michelle J. Bauer, Saleh F. Alquethamy, Scott A. Beatson, Jason A. Roberts, David L. Paterson, Alastair G. McEwan, Mark A. T. Blaskovich, Mark A. Schembri, Christopher A. McDevitt, Mark von Itzstein, Mark J. Walker

**Affiliations:** a The University of Queensland, School of Chemistry and Molecular Biosciences, Australian Infectious Diseases Research Centre, Brisbane, QLD, Australia; b The University of Queensland, UQ Centre for Clinical Research, Australian Infectious Diseases Research Centre, Brisbane, QLD, Australia; c The University of Queensland, Centre for Superbug Solutions, Institute for Molecular Bioscience, Brisbane, QLD, Australia; d Griffith University, Institute for Glycomics, Gold Coast, QLD, Australia; e The University of Melbourne, Department of Microbiology and Immunology, Peter Doherty Institute for Infection and Immunity, Melbourne, VIC, Australia; f Royal Brisbane and Women’s Hospital, Departments of Pharmacy and Intensive Care Medicine, Brisbane, QLD, Australia; g University of Montpellier, Division of Anaesthesiology Critical Care Emergency and Pain Medicine, Nîmes University Hospital, Nîmes, France; University of Oklahoma Health Sciences Center

**Keywords:** *Acinetobacter*, antibiotic resistance, ionophores, tetracyclines

## Abstract

Acinetobacter baumannii causes high mortality in ventilator-associated pneumonia patients, and antibiotic treatment is compromised by multidrug-resistant strains resistant to β-lactams, carbapenems, cephalosporins, polymyxins, and tetracyclines. Among COVID-19 patients receiving ventilator support, a multidrug-resistant A. baumannii secondary infection is associated with a 2-fold increase in mortality. Here, we investigated the use of the 8-hydroxyquinoline ionophore PBT2 to break the resistance of A. baumannii to tetracycline class antibiotics. *In vitro*, the combination of PBT2 and zinc with either tetracycline, doxycycline, or tigecycline was shown to be bactericidal against multidrug-resistant A. baumannii, and any resistance that did arise imposed a fitness cost. PBT2 and zinc disrupted metal ion homeostasis in A. baumannii, increasing cellular zinc and copper while decreasing magnesium accumulation. Using a murine model of pulmonary infection, treatment with PBT2 in combination with tetracycline or tigecycline proved efficacious against multidrug-resistant A. baumannii. These findings suggest that PBT2 may find utility as a resistance breaker to rescue the efficacy of tetracycline-class antibiotics commonly employed to treat multidrug-resistant A. baumannii infections.

## INTRODUCTION

Infections caused by carbapenem-resistant Acinetobacter baumannii are disseminated globally and are associated typically with high rates of morbidity and mortality (1). Habitually occurring in patients with significant health care system contact, A. baumannii is a major causative agent of bloodstream infections (BSIs), acute bacterial skin and skin structure infections (ABSSI), urinary tract infections, and ventilator-associated pneumonia (VAP). Within intensive care unit (ICU) settings, A. baumannii is one of the most common causative agents of VAP and is associated frequently with poor patient outcomes (2, 3). During the ongoing COVID-19 pandemic, challenges faced by health care professionals have been further exacerbated by multidrug-resistant (MDR) and extensively drug resistant (XDR) A. baumannii outbreaks (4–7). Recent reports suggest that up to 80% of ICU-admitted COVID-19 patients required invasive mechanical ventilation (8). Among these patients, MDR A. baumannii has been identified as a frequent cause of secondary bacterial infection, associated with a 2-fold increase in COVID-19-related mortality (9).

The persistence of A. baumannii in nosocomial settings has been attributed largely to the pathogen’s intrinsic ability to survive desiccation and exposure to disinfectants (10, 11). Moreover, A. baumannii demonstrates a high capability to develop resistance to multiple classes of antibiotics. In 2019, European resistance rates for invasive isolates resistant to three antimicrobial groups (fluoroquinolones, aminoglycosides, and carbapenems) exceeded 43% (12). In the United States during 2017, over 60% of isolates were resistant to all fluoroquinolones and extended-spectrum β-lactam class antibiotics, ampicillin-sulbactam combinations, and trimethoprim-sulfamethoxazole combinations (13). Treatment regimens encompassing broad-spectrum cephalosporins, β-lactam-β-lactamase inhibitor combinations, carbapenems, and tetracycline class antibiotics are common approaches used to treat antibiotic-susceptible A. baumannii infections (14–16). In the increasingly frequent scenario of resistance to the aforementioned agents, combination therapy incorporating tigecycline with the highly nephrotoxic antibiotic colistin remains a last-resort treatment option, to which resistance is now being reported (12, 17). The inability to provide patients with an adequate treatment strategy reduces the efficacy of patient care (13, 18).

We have reported previously that dysregulation of bacterial metal ion homeostasis breaks antibiotic resistance in select bacterial pathogens (19–21). Here, using the 8-hydroxyquinoline analog PBT2 [2-(dimethylamino) methyl-5,7-dichloro-8-hydroxyquinoline] (22, 23), we demonstrate that the disruption of metal homeostasis breaks resistance to tetracycline class antibiotics in carbapenem-resistant, MDR, and XDR A. baumannii clinical isolates *in vivo*, using murine wound and pulmonary infection models. PBT2 is an orally bioavailable hydroxyquinoline ionophore which mediates the transfer of zinc across biological membranes. Phase 2 clinical trials on PBT2 have been completed for neurodegenerative conditions in which once-daily oral dosing of 250 mg was generally safe and well tolerated when administered for up to 12 months (EURO, REACH, and IMAGINE clinical trials) (23–25). This study demonstrates the rescue of tetracycline class antibiotics, including tigecycline, which are commonly used to treat carbapenem-resistant A. baumannii infections.

## RESULTS

Monotherapy or combination therapy with next-generation tetracycline class antibiotics (e.g., tigecycline) is often employed as a last-resort measure to treat MDR and XDR A. baumannii infections. Unfortunately, resistance to these antibiotics is now being reported, which significantly reduces patient treatment options (26, 27). To address this challenge, in accordance with the Clinical and Laboratory Standards Institute (CLSI) and the European Committee on Antimicrobial Susceptibility Testing (EUCAST) guidelines (28, 29), the combination of PBT2 ± zinc to disrupt resistance to tetracycline class antibiotics in the clinical MDR A. baumannii strains MS14413 (30), AB5075 (31), and PGC-204089 (clinical isolate from the Royal Brisbane Women’s Hospital) and the XDR A. baumannii strain AB0057 (32) was investigated. All A. baumannii strains demonstrated resistance to either tetracycline, doxycycline, or tigecycline (Table 1). Upon addition of PBT2, susceptibility to tigecycline was observed for MS14413, AB0057, and PGC-204089; susceptibility to doxycycline was observed for MS14413 and PGC-204089; and susceptibility to tetracycline was observed for AB5075. Moreover, the addition of both PBT2 and zinc potentiated susceptibility, according to EUCAST breakpoints, to tetracycline, doxycycline, and tigecycline for MS14413, AB0057, PGC-204089, and AB5075 (Table 1).

**TABLE 1:** PBT2 and zinc resensitize tetracycline-resistant MDR and XDR A. baumannii strains to tetracycline class antibiotics^*a*^

Tetracycline class antibiotic	MIC (μg/mL) by A. baumannii strain and treatment															
	MS14413				AB0057				PGC-204089				AB5075			
	PBT2, 0 μM; Zn, 0 μM	PBT2, 64 μM; Zn, 0 μM	PBT2, 0 μM; Zn, 8 μM	PBT2, 64 μM; Zn, 8 μM	PBT2, 0 μM; Zn, 0 μM	PBT2, 64 μM; Zn, 0 μM	PBT2, 0 μM; Zn, 32 μM	PBT2, 64 μM; Zn, 32 μM	PBT2, 0 μM; Zn, 0 μM	PBT2, 64 μM; Zn, 0 μM	PBT2, 0 μM; Zn, 8 μM	PBT2, 64 μM; Zn, 8 μM	PBT2, 0 μM; Zn, 0 μM	PBT2, 64 μM; Zn, 0 μM	PBT2, 0 μM; Zn, 32 μM	PBT2, 64 μM; Zn, 32 μM
Tetracycline	16	4–8	16	**2**	>128	16	>128	**1**	64	32	64	**2**	8	**1**	8	**0.5**
Doxycycline	4	**0.25**	4	**0.125**	16	8	16	**2**	4	**2**	4	**1**	**0.5**	**≤0.125**	**0.5**	**≤0.125**
Tigecycline	8	**0.5**	8	**0.25**	8	**2**	8	**2**	4	**1**	4	**0.5**	**1**	**≤0.125**	**1**	**≤0.125**

a Resistance to tetracycline, doxycycline, and tigecycline was assessed for MDR A. baumannii strains MS14413, PGC-204089, and AB5075 and XDR A. baumannii strain AB0057. MIC assays were undertaken in the absence (untreated) or presence of PBT2, zinc, or PBT2 + zinc. MIC values highlighted in bold indicate an antibiotic susceptible breakpoint (≤2 μg/ml) in accordance with EUCAST guidelines for antimicrobial sensitivity testing. Data represent the mean of 3 biological replicates (29).

**FIG 1:**
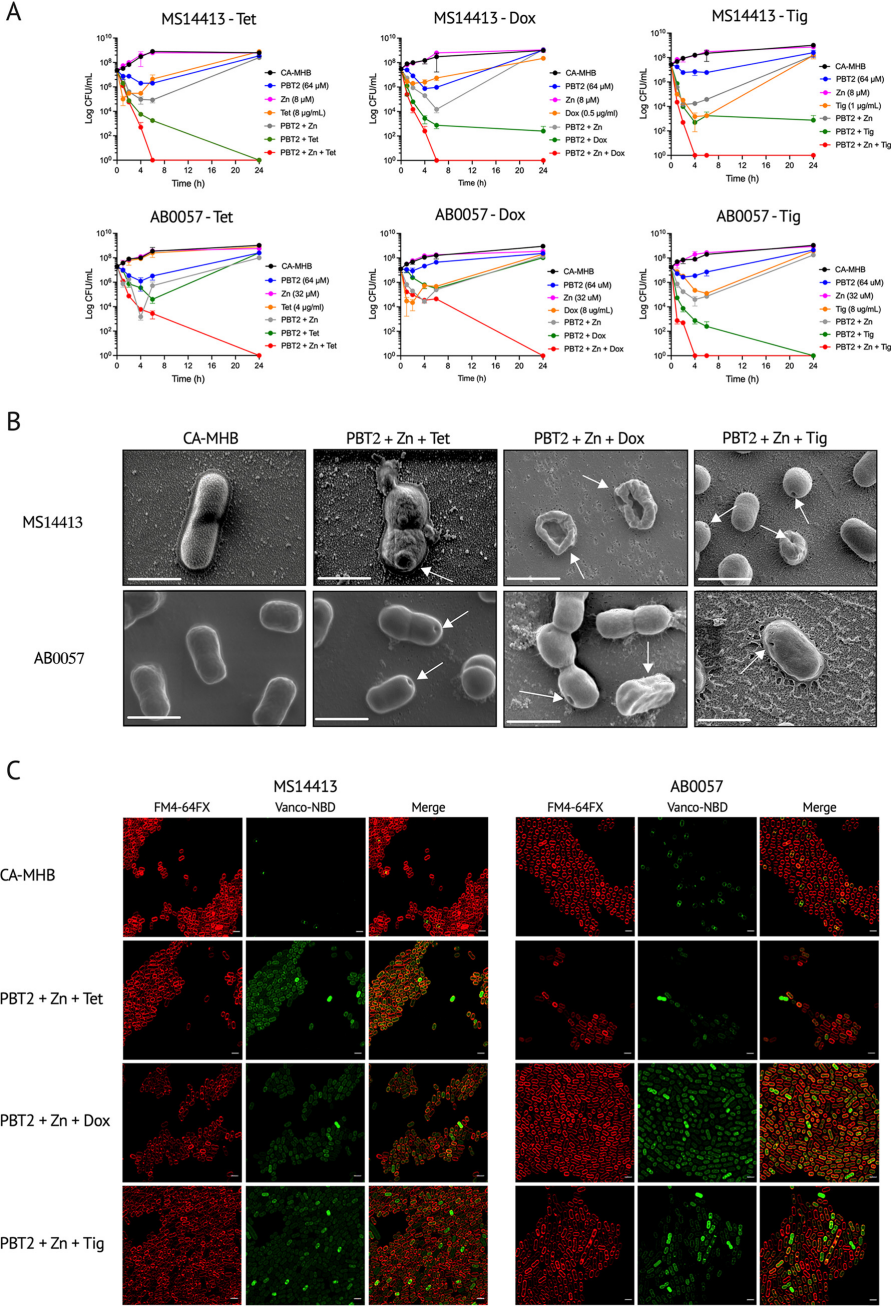
PBT2 + zinc in combination with tetracycline class antibiotics induces a bactericidal effect against tetracycline-resistant A. baumannii. (A) Time-kill curves for MDR A. baumannii strain MS14413 and XDR A. baumannii strain AB0057 in CA-MHB with or without PBT2, zinc, and tetracycline class antibiotics. Error bars indicate standard deviation from three biological replicates. (B) Scanning electron microscopy images of A. baumannii strains MS14413 and AB0057 grown in CA-MHB or in CA-MHB supplemented with PBT2 (64 μM) and zinc (8 μM for MS14413; 32 μM for AB0057) and either tetracycline (8 μg/mL for MS14413; 4 μg/mL for AB0057), doxycycline (0.5 μg/mL for MS14413; 8 μg/mL for AB0057), or tigecycline (1 μg/mL for MS14413; 8 μg/mL for AB0057) for 24 h at 37°C. Scale bars = 1 μm. Arrows indicate membrane indentations and membrane ruffling. (C) Confocal microscopy analysis of A. baumannii strains MS14413 and AB0057 grown in CA-MHB or in CA-MHB supplemented with PBT2 (64 μM) and zinc (8 μM for MS14413; 32 μM for AB0057) and either tetracycline (8 μg/mL for MS14413; 4 μg/mL for AB0057), doxycycline (0.5 μg/mL for MS14413; 8 μg/mL for AB0057), or tigecycline (1 μg/mL for MS14413; 8 μg/mL for AB0057) for 8 h at 37°C. Changes in bacterial membrane permeability were detected by staining bacteria with membrane-impermeable vancomycin-NBD fluorescent probe and membrane-labeling FM 4-64FX dye. Scale bars = 2 μm.

As shown by the treatment of A. baumannii strains MS14413 and AB0057, the combination PBT2 + zinc in the presence of either tetracycline, doxycycline, or tigecycline was observed to be bactericidal, indicated by a >3-log reduction in viable bacteria over 24 h (Fig. 1A). Furthermore, PBT2 alone and PBT2 + zinc visibly altered bacterial cell morphology, increasing the membrane permeability properties of both A. baumannii MS14413 and AB0057, respectively (Fig. 1B and C; see Fig. S1 and S2 in the supplemental material). As evidenced through visible membrane indentations and bacterial cell rupture, these effects were further augmented by the presence of either tetracycline, doxycycline, or tigecycline (Fig. 1B; Fig. S1).

10.1128/mbio.03517-21.1 FIG S1 Tetracycline class antibiotics in the presence of PBT2 and zinc alter bacterial cell morphology. Scanning electron microscopy images of A. baumannii strains MS14413 and AB0057 grown in CA-MHB or in CA-MHB supplemented with combinations of PBT2 (64 μM) and zinc (8 μM for MS14413; 32 μM for AB0057) and either tetracycline (8 μg/mL for MS14413; 4 μg/mL for AB0057), doxycycline (0.5 μg/mL for MS14413; 8 μg/mL for AB0057), or tigecycline (1 μg/mL for MS14413; 8 μg/mL for AB0057) for 24 h at 37°C. Scale bars = 1 μm. Arrows indicate membrane indentations and membrane ruffling. Download FIG S1, PDF file, 0.5 MB. Copyright © 2022 De Oliveira et al. 2022 De Oliveira et al. https://creativecommons.org/licenses/by/4.0/ This content is distributed under the terms of the Creative Commons Attribution 4.0 International license.

10.1128/mbio.03517-21.2 FIG S2 PBT2 and zinc affect the membrane permeability properties of A. baumannii. (A) Confocal microscopy images of A. baumannii strains MS14413 and AB0057 grown in CA-MHB or in CA-MHB supplemented with PBT2 (64 μM) and zinc (8 μM for MS14413; 32 μM for AB0057) and either tetracycline (8 μg/mL for MS14413; 4 μg/mL for AB0057), doxycycline (0.5 μg/mL for MS14413; 8 μg/mL for AB0057), or tigecycline (1 μg/mL for MS14413; 8 μg/mL for AB0057) for 8 h at 37°C. Changes in bacterial membrane permeability were detected by staining bacteria with the membrane-impermeable vancomycin-NBD fluorescent probe and the membrane-labeling FM 4-64FX dye. Scale bars = 2 μm. (B) Flow cytometric analysis of A. baumannii strains MS14413 and AB0057 grown in CA-MHB or in CA-MHB supplemented with PBT2 (64 μM) and zinc (8 μM for MS14413; 32 μM for AB0057) and either tetracycline (Tet; 8 μg/mL for MS14413; 4 μg/mL for AB0057), doxycycline (Dox; 0.5 μg/mL for MS14413; 8 μg/mL for AB0057), or tigecycline (Tig; 1 μg/mL for MS14413; 8 μg/mL for AB0057) for 8 h at 37°C. Changes in bacterial membrane permeability were detected by staining bacteria with the membrane-impermeable vancomycin-NBD fluorescent probe. Data represent the mean ± SEM of at least three biological replicates (****, *P* ≤ 0.0001; one-way ANOVA). Download FIG S2, PDF file, 0.3 MB. Copyright © 2022 De Oliveira et al. 2022 De Oliveira et al. https://creativecommons.org/licenses/by/4.0/ This content is distributed under the terms of the Creative Commons Attribution 4.0 International license.

**FIG 2:**
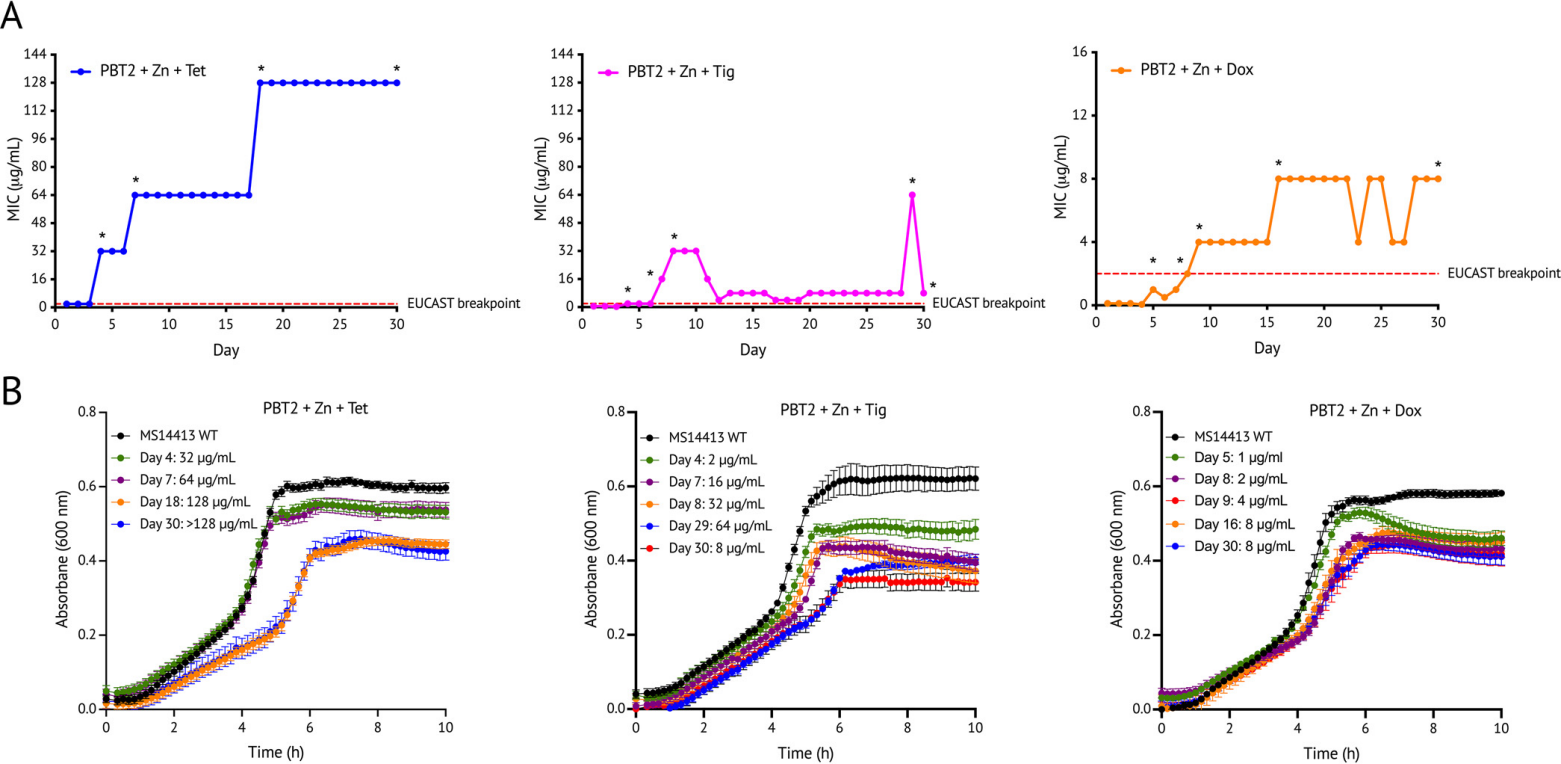
Resistance development against PBT2, zinc, and tetracycline class antibiotic combinations. (A) Development of resistance by A. baumannii strain MS14413 during serial passage with tetracycline, doxycycline, or tigecycline in the presence of subinhibitory concentrations of PBT2 and zinc in CA-MHB. Asterisks indicate time points where single colonies were isolated from a resistant culture. (B) Bacterial growth of resistant mutants (PBT2 + Zn + tetracycline class antibiotic) compared with that of wild-type (WT) A. baumannii MS14413. Growth curves are representative of three biological replicates.

We next investigated the likelihood of resistance development following treatment with tetracycline class antibiotics and PBT2 + zinc using the MDR A. baumannii strain MS14413. During serial passage for a period of 30 days in the presence of PBT2 + zinc and tetracycline class antibiotics, A. baumannii MS14413 demonstrated an appreciable increase in the MIC to tetracycline, doxycycline, and tigecycline, ranging from 31- to 127-fold (Fig. 2A). A 15-fold increase in the MIC of the control antibiotic rifampicin was also observed over the same time period (see Fig. S3 in the supplemental material). Compared with PBT2 in the presence of tetracycline or doxycycline, the reduced level of resistance for PBT2 in the presence tigecycline is possibly attributed to the presence of the *N*,*N*,-dimethylglycylamido side chain of tigecycline which has been associated with a decreased susceptibility to tigecycline resistance development (33). Notably, resistance to tetracycline, doxycycline, or tigecycline in the presence of PBT2 + zinc imposed a fitness cost, as evidenced by reduced growth in cation-adjusted Mueller-Hinton broth (CA-MHB) and lower survival in the lungs of mice compared with wild-type A. baumannii MS14413 (Fig. 2B; see Fig. S4 in the supplemental material). Whole-genome sequencing (WGS) of mutants resistant to tetracycline class antibiotics in the presence of PBT2 + zinc identified the presence of multiple chromosomal differences compared with the MS14413 reference genome (see Table S1 in the supplemental material). Notably, for mutants resistant to tetracycline in the presence of PBT2 + zinc, WGS identified the presence of insertion sequence (IS) IS*Aba125* in the TetR-family system regulator gene *adeN* which regulates the expression of the resistance-nodulation-cell division (RND) pathway AdeIJK (34, 35) (see Fig. S5 in the supplemental material; Table S1). Although the expression of AdeIJK contributes to the intrinsic resistance of A. baumannii to tetracycline class antibiotics, high levels of expression have been associated with toxicity to the bacterial cell (36). For mutants resistant to doxycycline in the presence of PBT2 + zinc, WGS identified appreciable stepwise mutations beginning in biofilm-associated cell-surface protein A (*bapA*) (37) followed by insertion of IS*Aba1* in *adeS* (encoding the AdeS sensor kinase of the two-component AdeRS regulatory system which regulates the RND AdeABC efflux pump system), proceeded by subsequent insertion of IS*Aba125* in *adeN* (Fig. S5; Table S1). Mutations in the two-component regulator AdeRS have been shown previously to result in an overexpression of the RND efflux system AdeABC, leading to moderate increases in resistance to tetracycline class antibiotics (38, 39). Observed changes in *bapA* are likely attributable to variation in the repetitive region. Although the impact of these changes has not been determined fully, they could potentially contribute to elevated MICs. For mutants resistant to tigecycline in the presence of PBT2 + zinc, WGS identified a stepwise insertion of IS*Aba125* in *adeN* followed by an insertion of IS*Aba1* in *adeS* (Fig. S5; Table S1). These mutations, combined with the previously noted chromosomal differences, may help explain the temporal stepwise development of resistance.

10.1128/mbio.03517-21.3 FIG S3 Resistance development to rifampicin. Development of resistance by A. baumannii strain MS14413 during serial passage with rifampicin in CA-MHB for 30 days. Download FIG S3, PDF file, 0.02 MB. Copyright © 2022 De Oliveira et al. 2022 De Oliveira et al. https://creativecommons.org/licenses/by/4.0/ This content is distributed under the terms of the Creative Commons Attribution 4.0 International license.

10.1128/mbio.03517-21.4 FIG S4 *In vivo* fitness of mutant A. baumannii resistant to PBT2 + tetracycline class antibiotics. Cohorts of BALB/C mice (*n* = 10) were challenged intranasally with MDR A. baumannii strain MS14413 (1.5 × 10^8^ CFU) or resistant A. baumannii MS14413 mutants isolated at day 30 of serial passage in subinhibitory concentrations of PBT2 + tetracycline (1.3 × 10^8^ CFU) or PBT2 + tigecycline (1.4 × 10^8^ CFU). CFUs were recovered 24 h postchallenge. Values for individual mice are plotted (*, *P* ≤ 0.05; ****, *P* ≤ 0.0001; one-way ANOVA with Tukey multiple comparisons). Download FIG S4, PDF file, 0.02 MB. Copyright © 2022 De Oliveira et al. 2022 De Oliveira et al. https://creativecommons.org/licenses/by/4.0/ This content is distributed under the terms of the Creative Commons Attribution 4.0 International license.

10.1128/mbio.03517-21.5 FIG S5 Insertion of IS*Aba125* in *adeN* and insertion IS*Aba1* in *adeS* may mediate resistance to tetracycline class antibiotics in the presence of PBT2 and zinc. (A) Whole-genome sequencing identified the insertion of IS*Aba125* in *adeN* and insertion of IS*Aba1* in the *adeS* gene of A. baumannii MS14413 mutants resistant to tetracycline class antibiotics (MIC of ≥2 μg/mL) in the presence of PBT2 and zinc. (B) PCR amplification of *adeN* and *adeS* genes in wild-type and mutant A. baumannii MS14413 resistant to tetracycline class antibiotics in the presence of PBT2 and zinc. PCR amplified products were electrophorized on a 1% agarose gel. Download FIG S5, PDF file, 0.3 MB. Copyright © 2022 De Oliveira et al. 2022 De Oliveira et al. https://creativecommons.org/licenses/by/4.0/ This content is distributed under the terms of the Creative Commons Attribution 4.0 International license.

10.1128/mbio.03517-21.8 TABLE S1 MS14413 mutant chromosomal differences as identified by Illumina whole-genome sequencing. Gray boxes indicate the presence of a chromosomal difference. Download Table S1, DOCX file, 0.04 MB. Copyright © 2022 De Oliveira et al. 2022 De Oliveira et al. https://creativecommons.org/licenses/by/4.0/ This content is distributed under the terms of the Creative Commons Attribution 4.0 International license.

**FIG 3:**
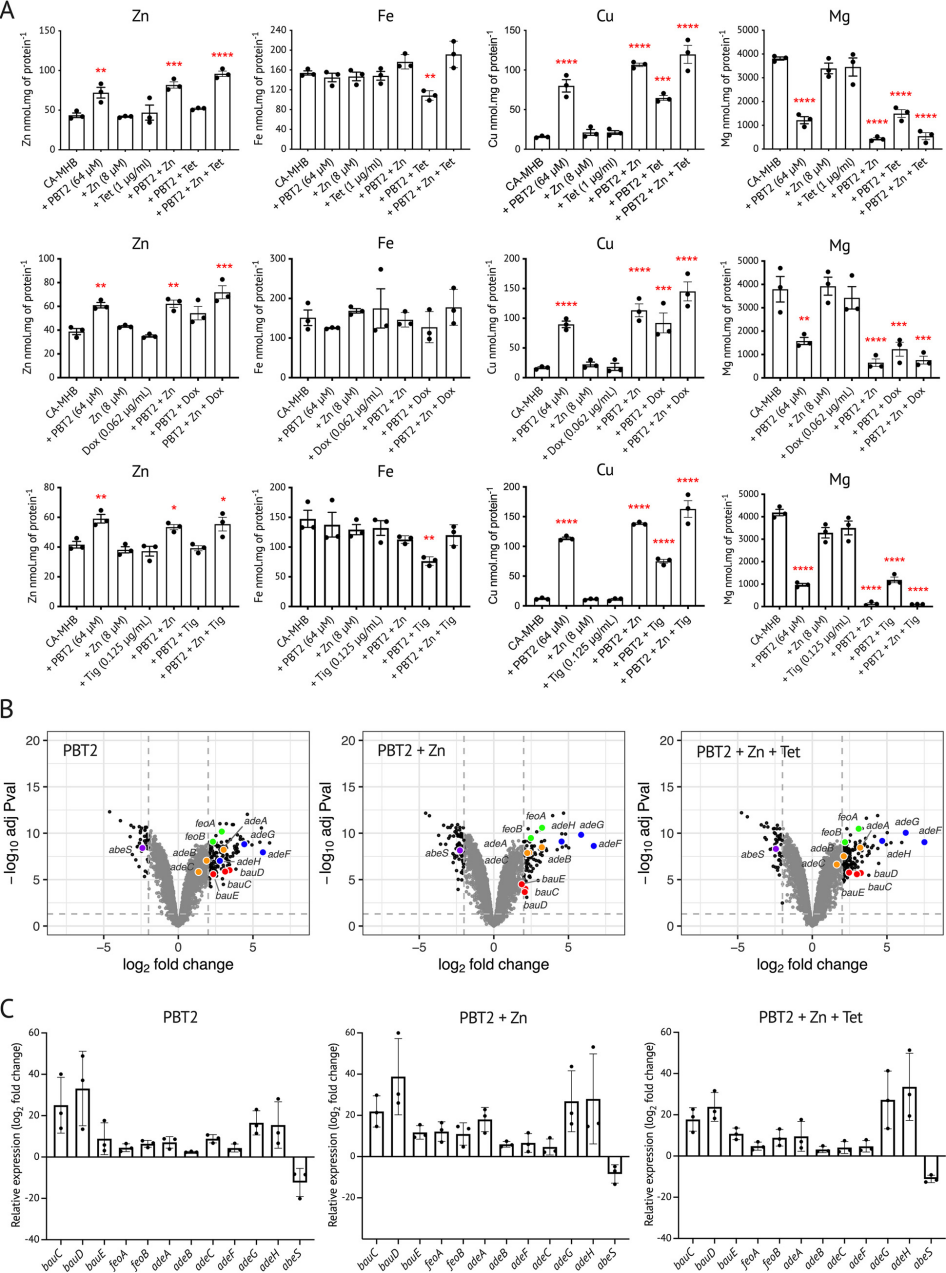
PBT2 dysregulates metal homeostasis in A. baumannii strain MS14413. (A) Whole cell zinc, iron, magnesium, and copper levels were assessed in A. baumannii strain MS14413 by inductively coupled plasma mass spectrometry. Bacteria were grown in CA-MHB in the absence or presence of PBT2, zinc, and tetracycline. Error bars indicate standard error of the mean from three biological replicates, *, *P* ≤ 0.05; **, *P* ≤ 0.01; ***, *P* ≤ 0.001; ****, *P* ≤ 0.0001; one-way ANOVA. (B) Volcano plots illustrating RNA-seq transcriptome analysis of A. baumannii strain MS14413 treated with either PBT2 (64 μM); PBT2 (64 μM) and zinc (8 μM); or PBT2 (64 μM), zinc (8 μM), and tetracycline (0.5 μg/mL) in CA-MHB. Genes with log_2_ fold change of >2 or <−2 and a *P* value of <0.05 are depicted by black dots; Feo system genes (*feoA* and *feoB*) are depicted by green dots; ferric acinetobactin ABC transporter system genes (*bauC*, *bauD*, *and bauE*) are depicted by red dots; multidrug efflux resistance nodulation division transport system genes (*adeABC* and *adeFGH*) are depicted by orange and blue dots, respectively; SMR family drug efflux system gene *abeS* is depicted by the purple dot. Data were collected from three biological replicates. (C) Transcript levels for selected genes measured by quantitative real-time PCR. Log_2_ fold changes were calculated relative to untreated controls and normalized to the A. baumannii reference gene *recA* using the ΔΔ*CT* method. Error bars represent standard deviation of the mean of three biological replicates.

PBT2 facilitates the direct permeation of zinc ions across biological membranes, independent of membrane protein-dependent transport pathways (19). PBT2 treatment of A. baumannii MS14413 increased the cellular content of zinc and copper and reduced magnesium. Notably, iron was not affected significantly (Fig. 3A). The effect of PBT2 on cellular copper was amplified by the presence of exogenous zinc, indicating broad metal ion dyshomeostatic impacts. Treatment of MS14413 with tetracycline class antibiotics alone had no direct effect on cellular metal content (Fig. 3A). To gain insight into the potential mechanism of action of PBT2 and zinc treatment, a transcriptome analysis of A. baumannii MS14413 was undertaken upon treatment with subinhibitory concentrations of PBT2, zinc, and tetracycline. Treatment with PBT2 was associated with changes in iron-specific metal stress response systems and drug efflux systems (Fig. 3B; see Table S2 in the supplemental material), which was confirmed by quantitative real-time PCR (Fig. 3C). Specifically, PBT2 led to the upregulation of iron uptake systems, including Feo system genes (*feoA* and *feoB*), ferric acinetobactin ABC transporter system genes (*bauC*, *bauD*, and *bauE*), and TonB receptor genes (*fpvA* and *pfeA*). Additionally, PBT2 exposure resulted in the upregulation of multidrug efflux resistance nodulation division transport system genes (*adeABC* and *adeFGH*) and downregulation of the small multidrug resistance (SMR) family drug efflux system gene *abeS*. No PBT2-dependent changes in the primary zinc, copper, or magnesium transport systems of A. baumannii were observed (40, 41) (Table S2). Nevertheless, the putative zinc exporting cation diffusion facilitator (CDF) gene *czcE* was upregulated suggesting zinc intoxication may be sensed by the bacterium (Table S2). These transcriptional changes were PBT2 dependent and were not affected by the presence of zinc or tetracycline (see Fig. S6 in the supplemental material; Table S2).

10.1128/mbio.03517-21.6 FIG S6 RNA transcriptome analysis of A. baumannii strain MS14413 in response to treatment with PBT2, zinc, and tetracycline. Volcano plot illustrates comparative RNA-seq analysis of A. baumannii MS14413 treated with PBT2 (64 μM) against treatment with PBT2 and zinc (8 μM) or treatment with PBT2, zinc, and tetracycline (1 μg/mL) in CA-MHB. Data were collected from three biological replicates. Download FIG S6, PDF file, 0.5 MB. Copyright © 2022 De Oliveira et al. 2022 De Oliveira et al. https://creativecommons.org/licenses/by/4.0/ This content is distributed under the terms of the Creative Commons Attribution 4.0 International license.

10.1128/mbio.03517-21.9 TABLE S2 A. baumannii MS14413 upregulated genes following 1-h treatment with 64 μM PBT2 in CA-MHB grown at 37°C. A. baumannii MS14413 downregulated genes following 1-h treatment with 64 μM PBT2 in CA-MHB grown at 37°C. A. baumannii MS14413 upregulated genes following 1-h treatment with 64 μM PBT2 and 8 μM ZnSO_4_ in CA-MHB grown at 37°C. A. baumannii MS14413 downregulated genes following 1-h treatment with 64 μM PBT2 and 8 μM ZnSO_4_ in CA-MHB grown at 37°C. A. baumannii MS14413 upregulated genes following 1-h treatment with 64 μM PBT2, 8 μM ZnSO_4_, and 1 μg/mL tetracycline in CA-MHB grown at 37°C. A. baumannii MS14413 downregulated genes following 1-h treatment with 64 μM PBT2, 8 μM ZnSO_4_, and 1 μg/mL tetracycline in CA-MHB grown at 37°C. Download Table S2, DOCX file, 0.07 MB. Copyright © 2022 De Oliveira et al. 2022 De Oliveira et al. https://creativecommons.org/licenses/by/4.0/ This content is distributed under the terms of the Creative Commons Attribution 4.0 International license.

**FIG 4:**
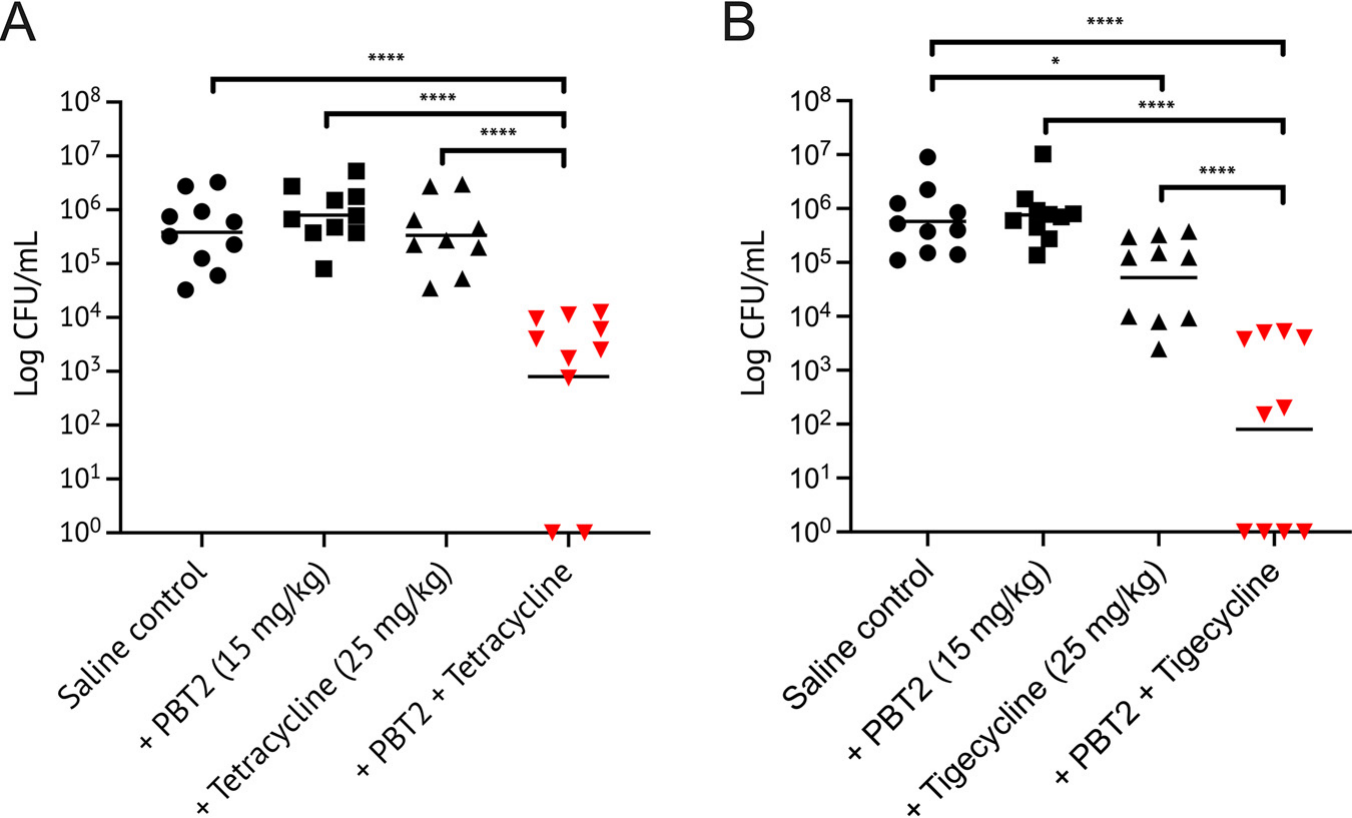
PBT2 breaks resistance to tetracycline class antibiotics in a pulmonary infection model. Cohorts of BALB/c mice (*n *= 10) were challenged intranasally with 1 × 10^8^ CFU of MDR A. baumannii strain MS14413. CFUs were recovered from the lungs 24 h postchallenge. Mice were treated with combinations of PBT2 (15 mg/kg; oral gavage) and tetracycline (25 mg/kg; intraperitoneal injection) (A) or PBT2 (15 mg/kg; oral gavage) and tigecycline (25 mg/kg; intraperitoneal injection) (B) at 0 h and 6 h postinfection. Values for individual mice are plotted (*, *P* ≤ 0.05; ****, *P* ≤ 0.0001; one-way ANOVA with Tukey multiple comparisons).

A. baumannii is a common cause of nosocomial ABSSI and VAP infections. An increase in the abundance of endogenous zinc at the site of infection is an important marker of bacterial wounds and lung infections (42–45). As such, we investigated the therapeutic potential of PBT2, in the absence of exogenous zinc, to break the resistance of A. baumannii MS14413 to tetracycline class antibiotics *in vivo.* Using a murine model of ABSSI, treatment with PBT2, tetracycline, or tigecycline alone had no therapeutic effect. A significant therapeutic reduction in bacterial burden at the site of infection was observed only when PBT2 was used in combination with tetracycline or tigecycline (see Fig. S7 in the supplemental material). For lung infection, PBT2 treatment alone had no therapeutic effect, while tigecycline alone provided some therapeutic benefit. The combination treatment of PBT2 and tetracycline, or PBT2 and tigecycline, abrogated infection-associated weight loss in mice and resulted in significant 2.5-log and 4-log reductions in the bacterial burden in the lungs, respectively (Fig. S7; Fig. 4). Collectively, these data suggest that PBT2 may sequester zinc from the host niche, facilitating the rescue of tetracycline class antibiotics.

10.1128/mbio.03517-21.7 FIG S7 PBT2 breaks resistance to tetracycline class antibiotics in a wound infection model and reduces infection-associated weight loss during murine pulmonary infection. For wound infection, CFUs were recovered from cohorts of BALB/C mice (*n *= 10) 4 days after challenge with 1 × 10^6^ CFUs of A. baumannii MS14413. Mice were treated twice daily with combinations of PBT2 (15 mg/kg; oral gavage) and tetracycline (25 mg/kg; intraperitoneal injection) (A) or combinations of PBT2 (15 mg/kg; oral gavage) and tigecycline (25 mg/kg; intraperitoneal injection) (B). Values for individual mice are plotted (**, *P* ≤ 0.01; ***, *P* ≤ 0.001; ****, *P* ≤ 0.0001; one-way ANOVA with Tukey multiple comparisons). For pulmonary infection, cohorts of BALB/C mice (*n* = 5) were challenged intranasally with 9.2 × 10^7^ CFUs of MDR A. baumannii strain MS14413. Mice were treated with combinations of PBT2 (15 mg/kg; oral gavage) and tetracycline (25 mg/kg; intraperitoneal injection) (C) or PBT2 (15 mg/kg; oral gavage) and tigecycline (25 mg/kg; intraperitoneal injection) (D) at 0 h and 6 h post infection. Mouse weight was measured at 0 h, 24 h, and 48 h postinfection. (*, *P* ≤ 0.05; one-way ANOVA with Tukey multiple comparisons). Download FIG S7, PDF file, 0.04 MB. Copyright © 2022 De Oliveira et al. 2022 De Oliveira et al. https://creativecommons.org/licenses/by/4.0/ This content is distributed under the terms of the Creative Commons Attribution 4.0 International license.

## DISCUSSION

Carbapenem-resistant A. baumannii (CRAB) is a worldwide health concern, and CRAB resistance to last-resort therapies (i.e., polymyxin and tigecycline combination/monotherapy) has been reported (12, 17). Since 2018, only two new therapies have been approved by the U.S. Food and Drug Administration and/or the European Union European Medicines Agency for the treatment of CRAB infection, i.e., cefiderocol and eravacycline (1). Unfortunately, cefiderocol-nonsusceptible strains of CRAB emerged prior to initial U.S. FDA approval (46). Moreover, eravacycline was approved specifically for intra-abdominal infection and not serious BSI and VAP infections (1). The lack of financial drivers for the development and commercialization of new antibiotic therapies often outweighs the value to public health, and therefore, no investments are made for new therapies (47). As such, rescuing the efficacy of existing therapies for the treatment of serious bacterial infection represents a financially viable pathway, reducing time, cost, and risk associated with drug innovation.

Antibiotic potentiator molecules represent a promising avenue for rescuing the efficacy of existing antimicrobial therapies (48). In complex with zinc, ionophore compounds have been shown previously to potentiate polymyxin and amikacin activity against A. baumannii *in vitro* (19, 49). Here, we demonstrate that the ionophore PBT2 breaks A. baumannii resistance to tetracycline class antibiotics *in vivo*. Our data indicate that PBT2 broadly perturbs A. baumannii metal ion homeostasis resulting in a redistribution of metal ions within the cell with elevated zinc and copper and decreased intracellular magnesium. This generalized dysregulation of metal homeostasis increases the efficacy of tetracycline class antibiotics. While zinc accumulation can be explained by PBT2 treatment, the mechanistic basis for the increased copper levels remains speculative. This increase could also be mediated by PBT2, but the upregulation of two *cntO* orthologs, the TonB receptors for secreted bacterial metallophores, may suggest an alternative pathway. Cnt-type systems function analogously with broad-spectrum metallophores to compete for essential metals during infection (50). Although the biosynthetic pathway for a broad-spectrum metallophore in A. baumannii has yet to be defined, copper accumulation could be facilitated by such a pathway. Irrespective of the precise mechanistic basis, zinc and copper accumulation did not result in upregulation of the primary efflux systems *czcABC* and *copA*, respectively (40, 41). However, an increased expression of the CDF transporter *czcE*, which has been shown to be highly upregulated in response to zinc intoxication (41), was observed. Taken together, these data suggest that while the bacterium may sense the increased abundance of zinc, the overall levels of both metals are within the buffering capacity of the cell and do not trigger derepression of the major efflux systems. An alternative explanation may be that the elevated zinc and copper levels are disrupting the metalloregulatory systems that control expression of the efflux pathways. The decrease in cellular magnesium also failed to activate a starvation response. Bacterial sensing of magnesium levels differs from that of zinc and copper, which is normally achieved by the concerted action of a two-component regulator to sense extracellular magnesium and riboswitches to sense cytoplasmic magnesium (51, 52). Here, the availability of magnesium in the CA-MHB medium and the increased abundance of other divalent cations within the bacterium may explain the failure of A. baumannii to accurately sense magnesium depletion. Magnesium is a requisite cofactor in a wide variety of enzymes, and its depletion or displacement in both proteins and lipid scaffolds of the outer membrane by the increased abundance of zinc or copper could severely impact cellular viability (53). Magnesium is known to play a key role in maintaining the structural integrity of the lipopolysaccharide outer membrane of Gram-negative bacteria (54, 55). Through electrostatic interaction, magnesium binds to anionic phosphate groups of the inner core, enabling structural integrity of the outer leaflet. Disruptions in these important electrostatic cross-link interactions have been shown to result in lipopolysaccharide (LPS) release and subsequent bacterial membrane rupture (54, 56). Here, we hypothesize the PBT2-mediated depletion of cellular magnesium content may contribute to increased membrane permeability and associated membrane defects.

Unexpectedly, iron import [the Fe(II) import system Feo and the biosynthetic pathways for the Fe(III) siderophore acinetobactin] was upregulated in response to PBT2 treatment. It is possible that the upregulation of iron acquisition systems may be caused by PBT2-mediated movement of iron out of the cell or possibly mediated by displacement from metalloprotein sites by zinc or copper. Alternatively, the elevated iron scavenging response may be attributed to inappropriate sensing by iron-responsive regulators due to zinc or copper mismetallation. Iron is a crucial component of heme and iron-sulfur clusters, which are critical for respiratory enzymes in the electron transport chain and is an essential cofactor in a number of other cellular processes (57). Since A. baumannii is a nonfermentative bacterium that relies on respiratory electron transport, perturbation of iron homeostasis would have profound effects on cell physiology and metabolism.

Clinical trials have demonstrated that PBT2 is safe and well tolerated during use in humans (24). Here, we provide evidence that PBT2 potentiates tetracycline-class antibiotics *in vivo* against MDR A. baumannii in a murine model of pulmonary infection. This study highlights the potential of PBT2 to be used in combination with tetracycline class antibiotics for the treatment of severe infection caused by drug-resistant A. baumannii.

## MATERIALS AND METHODS

### Materials.

Tetracycline (catalog [cat.] no. 37919), doxycycline (cat. no. D9891), tigecycline (cat. no. PZ0021), and rifampicin (cat. no. R3501) were purchased from Sigma-Aldrich. PBT2 was produced by chemical synthesis (58), and the purity of the final product was confirmed to be >95% by ^1^H and ^13^C nuclear magnetic resonance (NMR), as described previously (21).

### Bacterial strains, media, and growth conditions.

A. baumannii strains MS14413 (30) AB0057 (32), AB5075 (31), and PGC-204089 (kindly provided by David L. Paterson) were grown in cation-adjusted Mueller-Hinton broth (CA-MHB) (cat. no. 212322, Becton Dickson) as per CLSI guidelines (28). Bacterial CFU enumeration was carried out on Luria Bertani broth (LB) agar. Bacteria were grown routinely at 37°C under aerobic conditions.

### MIC determination.

MICs and MIC breakpoints were determined by broth microdilution in accordance with CLSI/EUCAST guidelines as described previously (21, 28, 29). MIC assays were carried out in biological triplicate.

### Bacterial time-kill assays.

Bacteria were grown to an optical density at 600 nm (OD_600_) of 0.4 in CA-MHB and treated with and without combinations of PBT2 (64 μM) and ZnSO_4_ (8 μM for MS14413; 32 μM for AB0057) and either tetracycline (8 μg/mL for MS14413; 4 μg/mL for AB0057), doxycycline (0.5 μg/mL for MS14413; 8 μg/mL for AB0057), or tigecycline (1 μg/mL for MS14413; 8 μg/mL for AB0057) for 24 h at 37°C. To determine the rate of bacterial killing, aliquots of bacterial suspension were removed at 0, 1, 2, 4, 6, and 24 h; serially diluted in phosphate-buffered saline (PBS); and plated onto LB agar plates. Time-kill assays were performed in biological triplicate.

### Scanning electron microscopy (SEM).

SEM studies were undertaken at the Centre for Microscopy and Microanalysis at the University of Queensland. Bacterial strains were cultured in CA-MHB to an OD_600_ of 0.4 and treated in the absence and presence of PBT2 (64 μM) and ZnSO_4_ (8 μM for MS14413; 32 μM for AB0057) and either tetracycline (8 μg/mL for MS14413; 4 μg/mL for AB0057), doxycycline (0.5 μg/mL for MS14413; 8 μg/mL for AB0057), or tigecycline (1 μg/mL for MS14413; 8 μg/mL for AB0057) for 24 h at 37°C. Bacteria were washed twice with PBS preceding glutaraldehyde fixation. Samples were then dehydrated, assisted with a Pelco biowave regimen, via a series of ethanol treatments (30% to 100% EtOH), with one treatment with 100% EtOH-hexamethyldisilazane (HMDS; 1:1), and finally two treatments with 100% HMDS. Samples were applied to coverslips coated with poly-l-lysine (1 mg/mL) before being air dried for 2 h. Coverslips were attached to 13-mm SEM stubs with double-sided carbon tabs, plasma cleaned for 10 min in an Evactron De-contaminator (XEI Scientific), and coated with two layers of platinum (first layer, 0° angle from above; second layer, 45° angle from above) using a Turbomolecular pumped coater (Quorum Tech) following the manufacturer’s instructions. Samples were imaged in a JEOl JSM 7100F or JEOl JSM 7800F field emission SEM at an accelerating voltage of 1 to 3 kV.

### Membrane permeability assay.

From overnight cultures, A. baumannii strains MS14413 and AB0057 were diluted into fresh CA-MHB medium to an OD_600_ of 0.4. Bacteria were then treated in the absence or presence of PBT2 (64 μM) and ZnSO_4_ (8 μM for MS14413; 32 μM for AB0057) with either tetracycline (8 μg/mL for MS14413; 4 μg/mL for AB0057), doxycycline (0.5 μg/mL for MS14413; 8 μg/mL for AB0057), or tigecycline (1 μg/mL for MS14413; 8 μg/mL for AB0057) for 8 h at 37°C. Approximately 4 × 10^8^ CFU cells were then collected by centrifugation (3,200 × *g*, 2 min), and 500 μL of vancomycin-7-nitrobenzofurazan (NBD) fluorescent probe (59, 60) at 32 μg/mL was added to each sample and incubated for 30 min at 37°C with shaking (180 rpm). Bacteria were then labeled with 500 μL of FM 4-64FX (Thermofisher; cat. no. F34653) at 5 μg/mL (5 min on ice). For confocal microscopy, samples were then embedded on 1% agarose pads, and samples were analyzed on an inverted LSM 880 Fast Airyscan instrument (63×/1.40 oil). For flow cytometric analysis, bacteria were then diluted with Hanks’ balanced salt solution (Gibco) and analyzed on a CytoFlex S flow cytometer (Beckman Coulter). A fluorescein isothiocyanate (FITC) channel (excitation, 488 nm; emission, 525/40 nm) was used for the fluorescence intensity measurement of the vancomycin-NBD fluorescent probe, and 40,000 events were acquired per sample.

### Resistance development studies.

The development of resistance to tetracycline class antibiotics in the presence of PBT2 was undertaken as described previously (21). Briefly, A. baumannii MS14413 was sequentially passaged in subinhibitory concentrations of PBT2 + tetracycline class antibiotics over 30 days in CA-MHB. As a control for resistance development, the antibiotic rifampicin was used. Initially, the MIC for PBT2 with or without antibiotic was determined by broth microdilution following CLSI guidelines in a microtiter plate. The highest antibiotic or PBT2 + antibiotic concentration that still showed growth after overnight incubation was diluted 1:250 into a new microtiter plate containing 2-fold dilutions of antibiotic or PBT2 + antibiotic. This procedure was repeated for 30 days.

### Genome sequencing analysis.

DNA was extracted from overnight cultures of wild-type and mutant A. baumannii isolates resistant to tetracycline, doxycycline, or tigecycline in the presence of PBT2 + zinc using the DNeasy blood and tissue kit (Qiagen) as per the manufacturer’s instructions. Paired-end Illumina DNA libraries were prepared using the Nextera DNA flex library prep kit (Illumina, Australia) with a modification of the starting input of 5 μL genomic DNA (gDNA) with samples of >20 ng/μL. WGS of pooled libraries was performed using the Illumina MiniSeq system and the high-output reagent kit (300 cycles). Illumina paired-end reads were assembled using SPAdes v3.14.1 (PMID 24093227) with default settings. Draft genome assemblies were ordered and orientated by aligning them to the complete genome of MS14413 using ragtag v1.1.0 (PMID 31661016). Pairwise alignments to MS14413 were visualized using the Artemis comparison tool (ACT) v18.1.0 (PMID 15976072). Whole-genome comparisons were then used to identify differences between wild-type and mutant MS14413 genomes. Insertion sequences were identified and annotated by a blastN comparison of draft genomes against the ISfinder database (PMID 16381877). Single nucleotide polymorphism (SNP) profiling and determination of core genome SNPs was undertaken by aligning the trimmed Illumina sequencing reads for each sample to the complete genome of wild-type strain MS14413 (GenBank accession CP054302) using Snippy v4.4.1 with a minimum read coverage of 10× and minimum base quality of 20 that is required for a site to be considered.

### Growth analysis.

Overnight cultures of wild-type A. baumannii MS14413 and A. baumannii MS14413 resistant to PBT2 + tetracycline class antibiotics were standardized to an OD_600_ of 0.01 in CA-MHB medium. Bacteria were grown in a 96-well plate at a final volume of 200 μL and measured at 600 nm using a FLUOstar Omega microplate reader (BMG Labtech) at 37°C with shaking at 250 rpm. Growth assays were performed in biological triplicates and measured in technical triplicates.

### PCR.

PCR amplification of *adeN* and *adeS* genes in A. baumannii MS14413 were carried out using primers provided in Table S3 in the supplemental material.

10.1128/mbio.03517-21.10 TABLE S3 Primers used in this study. Download Table S3, DOCX file, 0.01 MB. Copyright © 2022 De Oliveira et al. 2022 De Oliveira et al. https://creativecommons.org/licenses/by/4.0/ This content is distributed under the terms of the Creative Commons Attribution 4.0 International license.

### ICP-MS.

From overnight cultures, A. baumannii MS14413 was grown to an OD_600_ of 0.5 in CA-MHB. Cells were then treated with combinations of PBT2 (64 μM), ZnSO_4_ (8 μM), and tetracycline (1 μg/mL) for 1 h at 37°C. Cells were harvested, processed, and analyzed using an Agilent 8900 ICP-QQQ instrument as described previously (21).

### RNA isolation.

RNA was isolated using a RNeasy Plus kit (Qiagen) as described previously (19). Briefly, A. baumannii MS14413 was grown to an OD_600_ of 0.5 in CA-MHB. Cells were then treated in the absence and presence PBT2 (64 μM), ZnSO_4_ (8 μM), and tetracycline (1 μg/mL) for 1 h at 37°C. Two volumes of RNAprotect (Qiagen) were added to the cultures, and the samples were then centrifuged at 5,000 × *g* for 25 min at 4°C to pellet cells. RNA was isolated from the dry pellet as per the manufacturer’s instructions. To ensure complete removal of DNA, the RNA was then further purified using the Turbo DNA-free kit (Thermo Fisher Scientific) according to the manufacturer’s instructions.

### Transcriptome sequencing (RNA-seq) analysis.

RNA-seq analysis was performed at the Australian Genome Research Facility. The cDNA library was prepared and assessed as described previously (19). An average of 30 million reads per sample were generated, then trimmed using trimmomatic (v0.36), and mapped to the reference genome (A. baumannii MS14413 accession number NZ_CP054302) using bowtie2 (v2.3.4.2) with default parameters (–local –very-sensitive-local). Read counts were determined using featureCounts (Rsubread v1.28.1), and differential gene expression was analyzed with edgeR (v3.20.9) and limma-voom (v3.34.9) with the counts per minute (cpm) threshold set at 2.0. Figures were generated using ggplot2 (v3.3.0) and ggrepel (v0.8.2) packages in Rstudio (61).

### Quantitative real-time PCR.

Genes associated with heavy metal homeostasis were selected for quantitative real-time PCR analysis. Quantitative real-time PCR was carried out as described previously (19). Relative gene expression was calculated by the threshold cycle (ΔΔ*CT*) method using *recA* as the reference gene for A. baumannii MS14413. All experiments were done in biological triplicates and measured in technical triplicates. Primers used for quantitative real-time PCR are provided in Table S3.

### Murine wound infection model.

For wound infection, 7-week-old female BALB/c mice were prepared, anesthetized, and subjected to superficial scarification as described previously (21). For infection, A. baumannii MS14413 was cultured to mid-log phase in tryptic soy broth (TSB), and 1 × 10^6^ CFU of bacteria was applied onto the scarified tissue in a final volume of 10 μL. Mice cohorts (*n *= 10) were treated with combinations of PBT2 (15 mg/kg of body weight; oral gavage), tetracycline (25 mg/kg; intraperitoneal injection), tigecycline (25 mg/kg; intraperitoneal injection), or saline solution (0.9%, wt/vol; intraperitoneal injection and/or oral gavage) in a 100-μL volume. Treatments were administered at 1, 6, 24, and 30 h postinfection. After 2 days of treatment, the mice were euthanized, the skin was excised, and bacteria were enumerated as described previously (19). Statistical differences in CFU were determined by a one-way analysis of variance (ANOVA), with a *P* value of <0.05 considered statistically significant (GraphPad Prism 9).

### Murine pulmonary infection model.

For pulmonary infection, cohorts of 7-week-old female BALB/c mice (*n* = 10) were challenged intranasally with a 25-μl preparation of 1 × 10^8^ CFU of mid-log-phase A. baumannii MS14413 or mutant A. baumannii MS14413 resistant to PBT2 + tetracycline or PBT2 + tigecycline. For treatments, mice cohorts were treated with combinations of PBT2 (15 mg/kg; oral gavage), tetracycline (25 mg/kg; intraperitoneal injection), tigecycline (25 mg/kg; intraperitoneal injection), or saline solution (0.9% [wt/vol]; intraperitoneal injection and/or oral gavage) in a 100-μL volume. Treatments were administered at 0 and 6 h postinfection. At 24 h postinfection, mice were euthanized, and the lungs were harvested and washed in sterile PBS. To determine viable bacteria in the lungs, tissue was homogenized and plated as described previously.

### Ethics.

Animal experiments were performed according to the Australian code of practice for the care and use of animals for scientific purposes. Permission was obtained from the University of Queensland ethics committee (SCMB/AIBN/144/17).

### Statistical analysis.

All experiments were undertaken either in technical duplicate or triplicate, with no less than 3 biological replicates. Data are presented as means ± SD. Bacterial dissemination data from *in vivo* studies are presented as geometric means. To compare means between more than two groups, a one-way ANOVA with *post hoc* (Tukey) was conducted. Murine survival curves were analyzed using the Mantel-Cox log-rank test. Statistical analysis was performed using GraphPad Prism software v9. A *P* value of <0.05 was considered statistically significant.

### Data availability.

RNA-seq data sets generated and analyzed during the current study are available in the GEO repository (GSE175535). Genome data have been deposited to NCBI under BioProject PRJNA733838. Raw Illumina sequence read data have been deposited to the Sequence Read Archive (SRA) under the accessions SRR14695606, SRR14695607, SRR14695609 to SRR14695613, SRR14695615, SRR14695617, SRR14695618, and SRR14695620 to SRR14695625.
