# Supplementary figures and images for: Rescuing Tetracycline Class Antibiotics for the Treatment of Multidrug-Resistant Acinetobacter baumannii Pulmonary Infection
Source: mBio. 2022 Jan 11;13(1):e03517-21. doi: 10.1128/mbio.03517-21 (PMC8749419; doi:10.1128/mbio.03517-21)

# *A. baumannii* MS14413

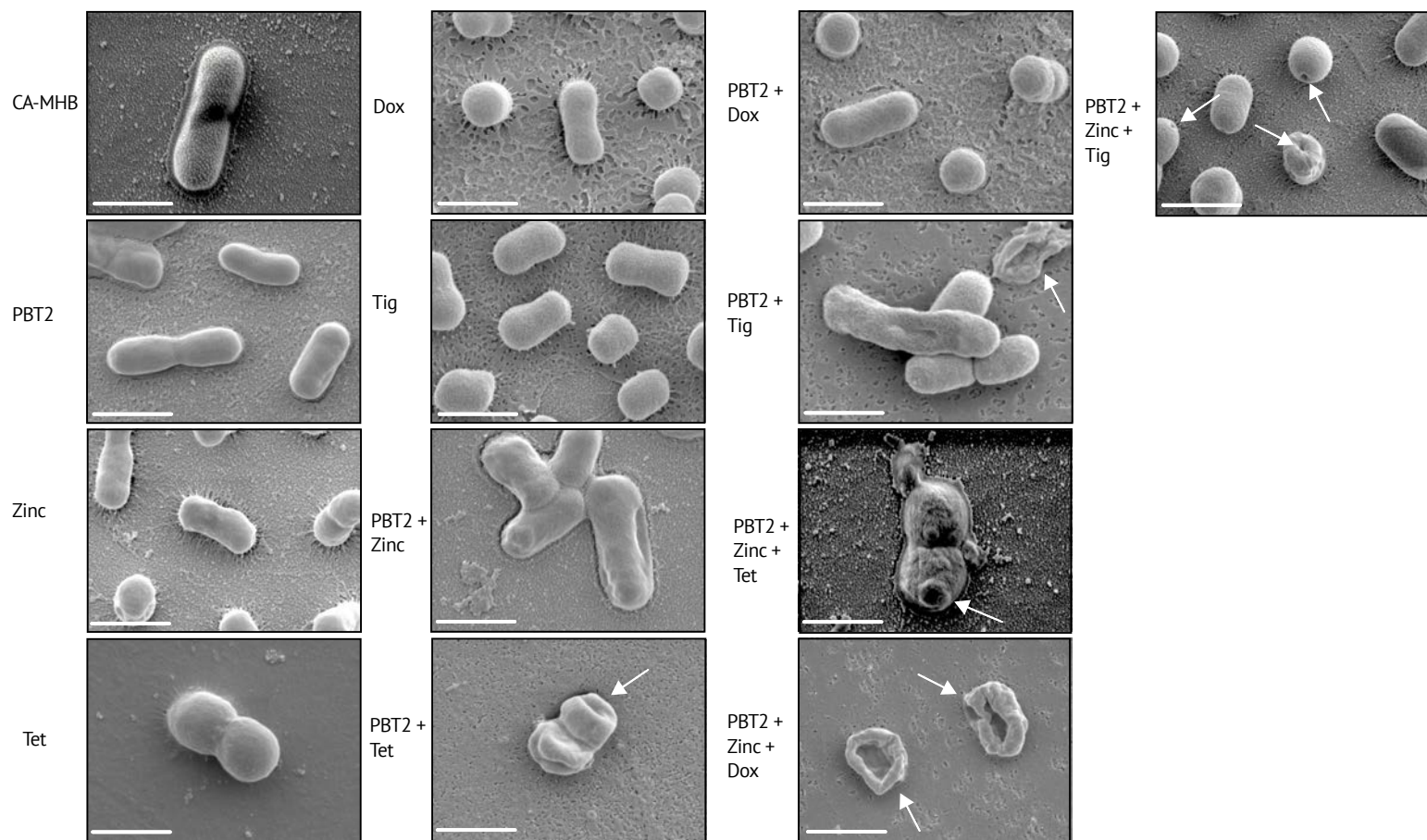

# *A. baumannii* AB0057

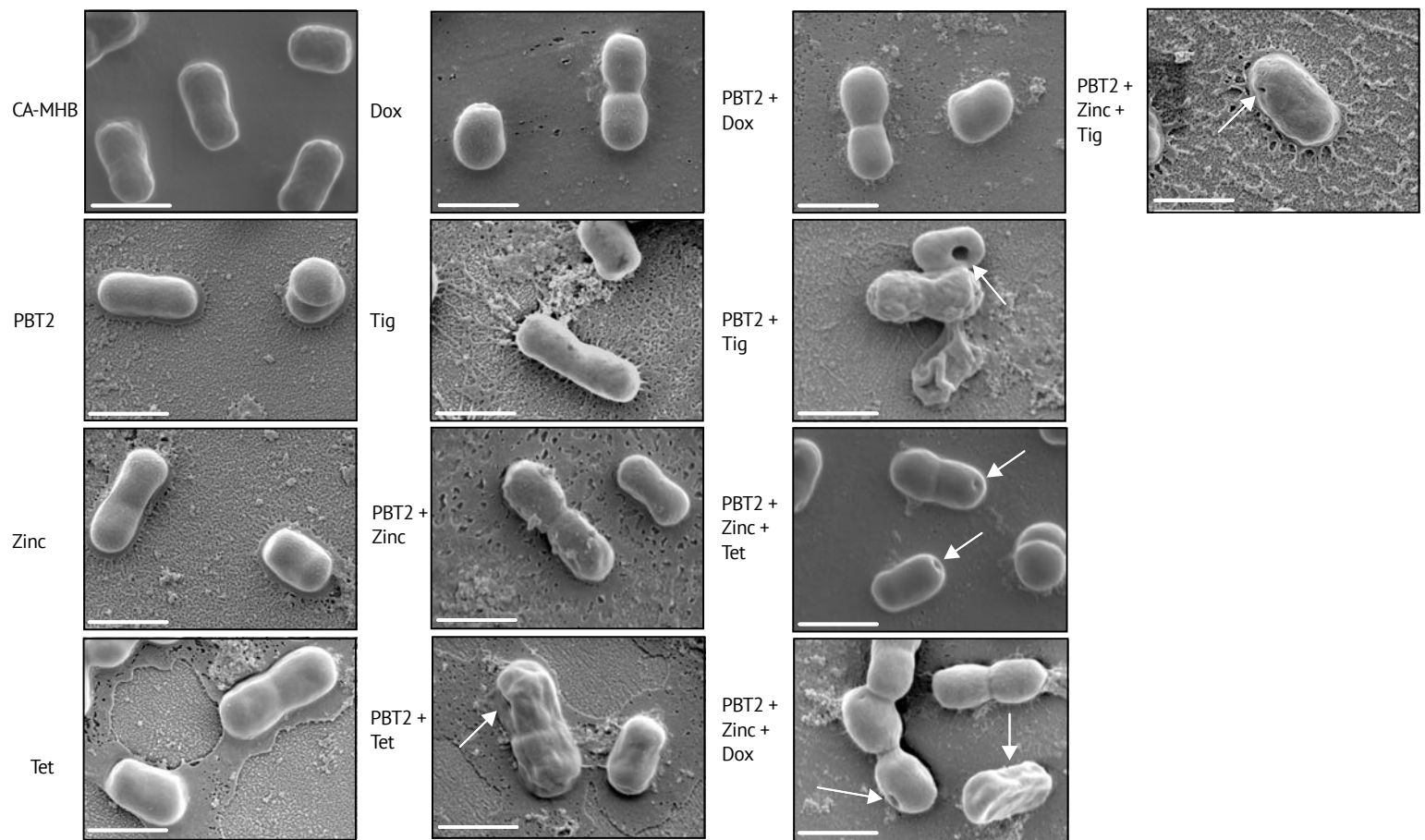

Supplement: FIG S1 [file mbio.03517-21-sf001.pdf]

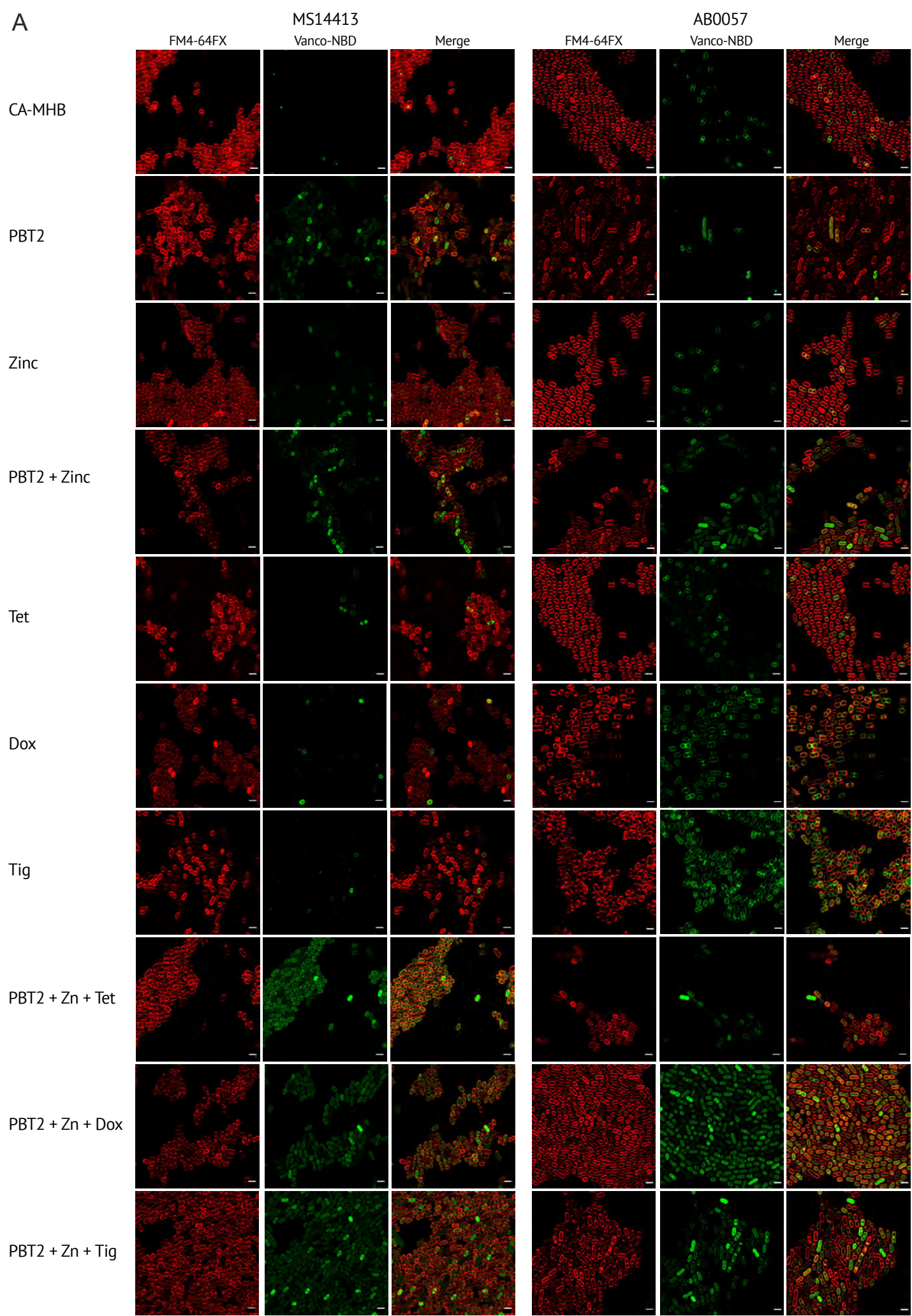

B

MS14413

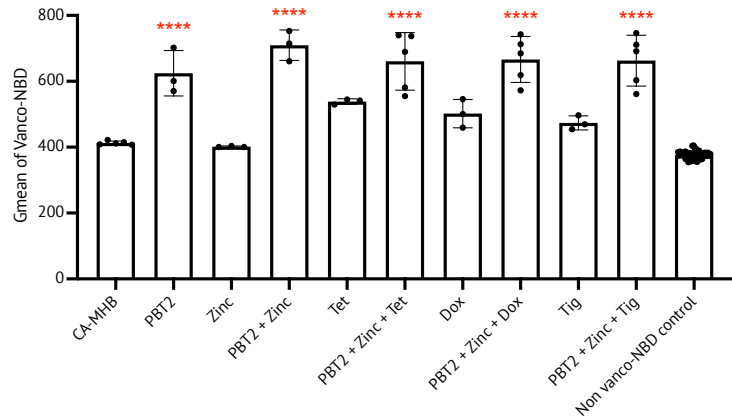

AB0057

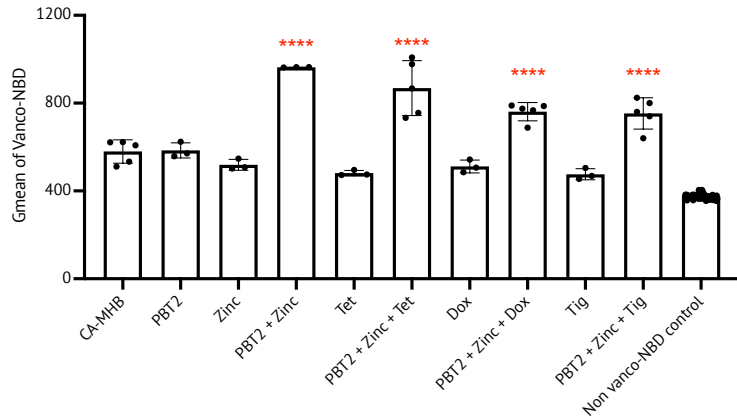

Supplement: FIG S2 [file mbio.03517-21-sf002.pdf]

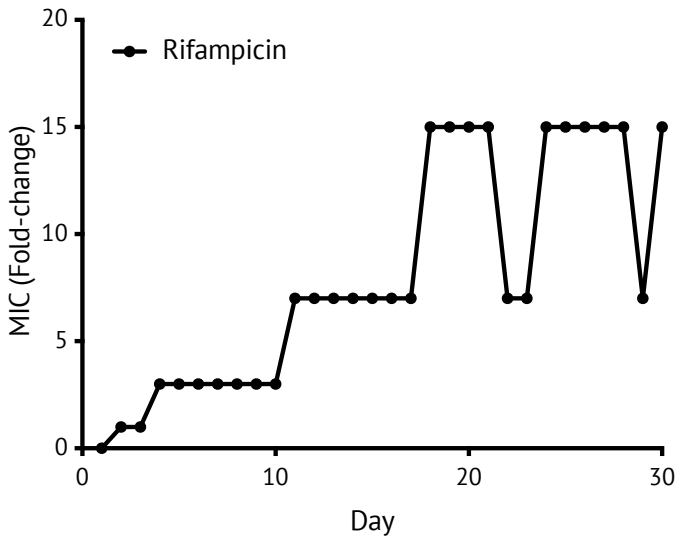

Supplement: FIG S3 [file mbio.03517-21-sf003.pdf]

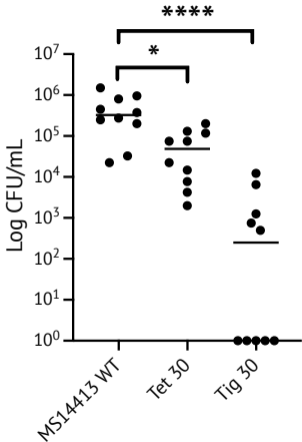

Supplement: FIG S4 [file mbio.03517-21-sf004.pdf]

A

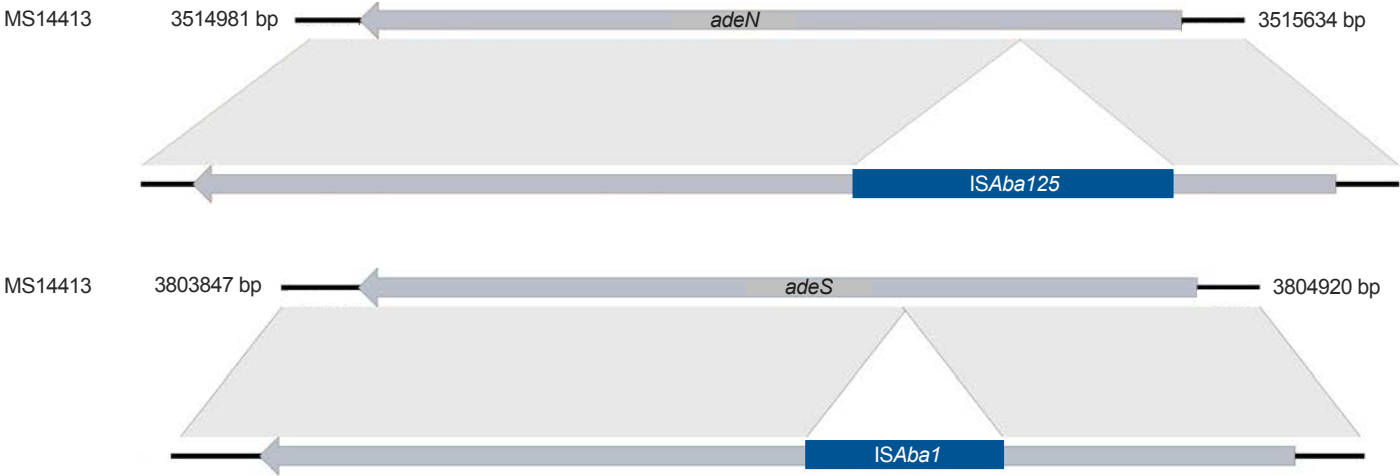

B

**Tetracycline**

**Doxycycline**

**Tigecycline**

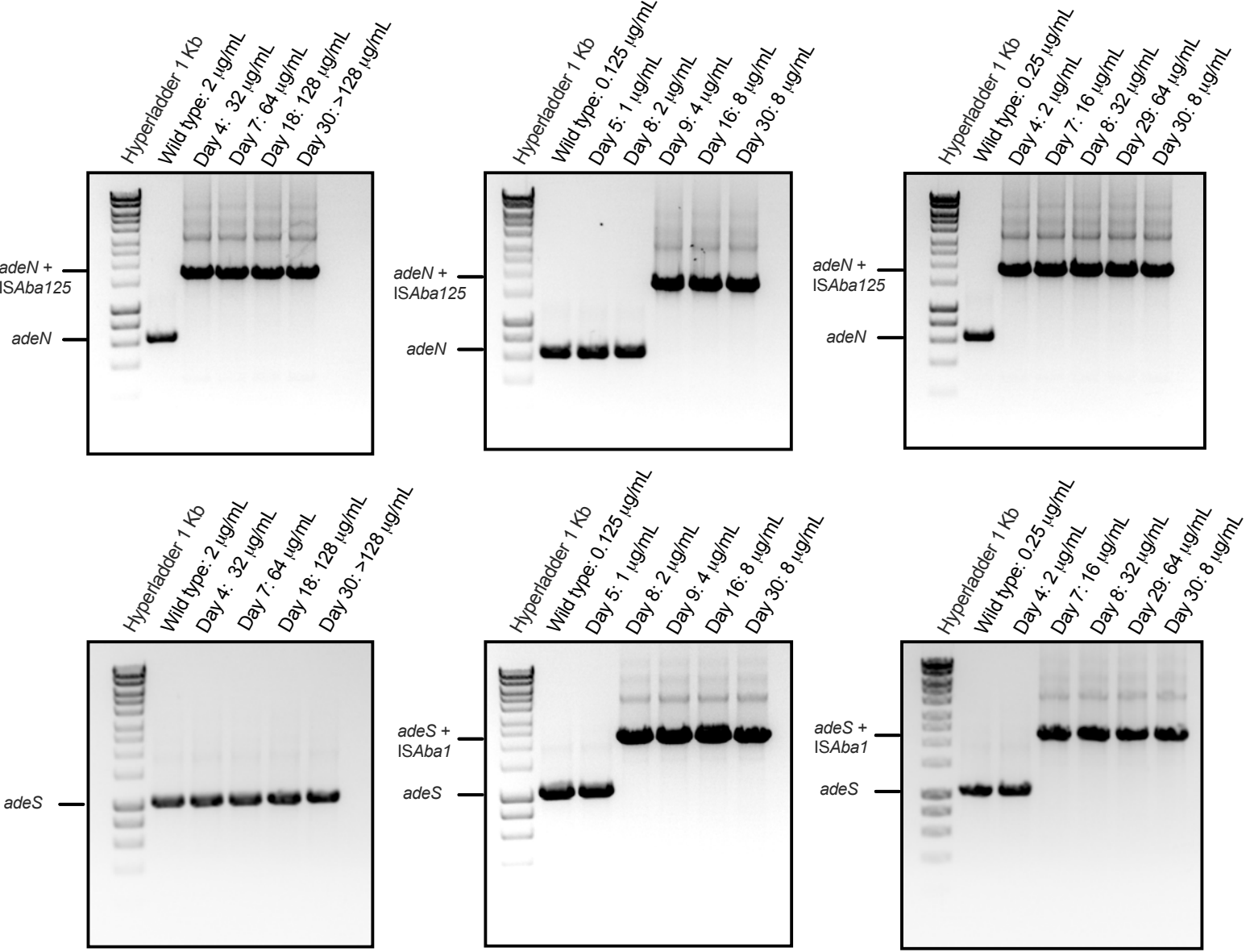

Supplement: FIG S5 [file mbio.03517-21-sf005.pdf]

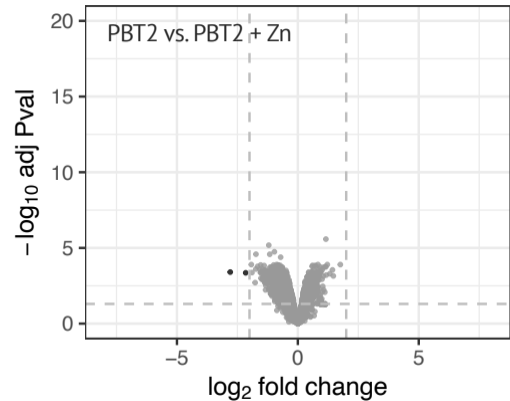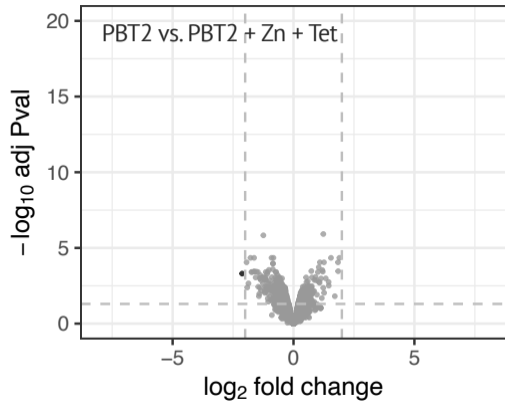

Supplement: FIG S6 [file mbio.03517-21-sf006.pdf]

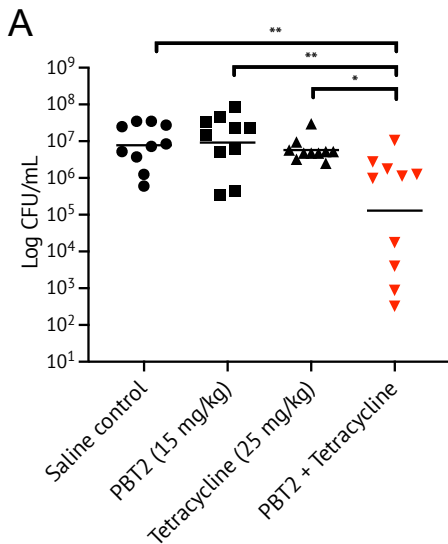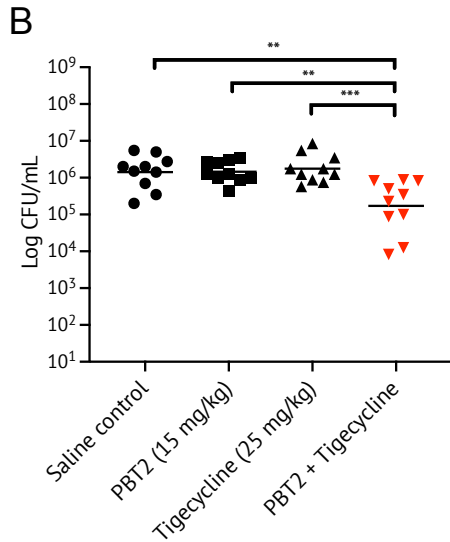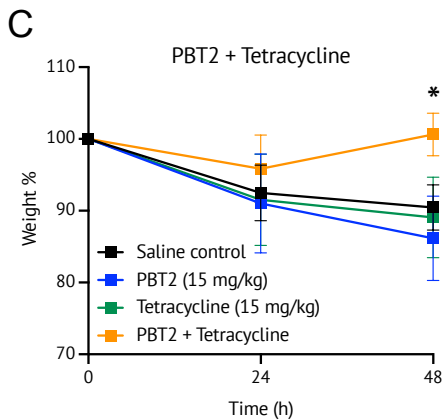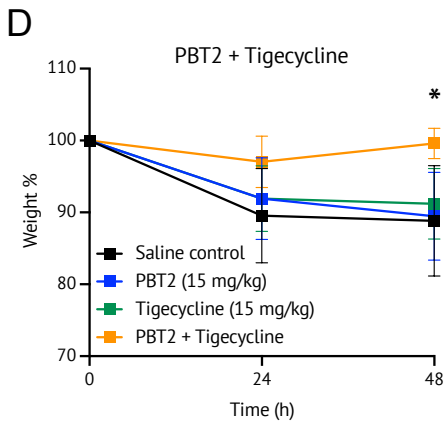

Supplement: FIG S7 [file mbio.03517-21-sf007.pdf]
