# Supplementary material for: Rescuing Tetracycline Class Antibiotics for the Treatment of Multidrug-Resistant Acinetobacter baumannii Pulmonary Infection
Source: mBio. 2022 Jan 11;13(1):e03517-21. doi: 10.1128/mbio.03517-21 (PMC8749419; doi:10.1128/mbio.03517-21)
Supplement: TABLE S1 [file mbio.03517-21-st001.docx]

**TABLE S1** MS14413 mutant chromosomal differences as identified by Illumina whole genome sequencing. Grey boxes indicate the presence of a chromosomal difference.

|  | | | | **Mutant isolate** | | | | | | | | | | | | | | |
| --- | --- | --- | --- | --- | --- | --- | --- | --- | --- | --- | --- | --- | --- | --- | --- | --- | --- | --- |
|  |  |  |  | **Tet**  **day 4** | **Tet**  **day 7** | | **Tet**  **day 18** | **Tet**  **day 30** | **Dox day 5** | **Dox day 8** | **Dox day 9** | **Dox day 16** | **Dox day 30** | **Tig day 4** | **Tig day 7** | **Tig day 8** | **Tig day 29** | **Tig day 30** |
|  | | | | **Mutant MIC (µg/ml)** | | | | | | | | | | | | | | |
| **Locus_tag** | **Gene; Product** | **Site_start** | **Site_end** | **32** | | **64** | **128** | **>128** | **1** | **2** | **4** | **8** | **8** | **2** | **16** | **32** | **64** | **8** |
| MS14413_00002 | *metK;* S-adenosylmethionine synthase | 3127 | 3199 |  | |  |  |  |  |  |  |  |  |  |  |  |  |  |
| MS14413_00021 | *eamA;* drug metabolite family transporter protein | 19299 | 19352 |  | |  |  |  |  |  |  |  |  |  |  |  |  |  |
| MS14413_00066 | *armA*; RNA methyltransferase | 66402 | 66457 |  | |  |  |  |  |  |  |  |  |  |  |  |  |  |
| MS14413_00138 | KGG domain-containing protein | 139283 | 139308 |  | |  |  |  |  |  |  |  |  |  |  |  |  |  |
| Intergenic | - | 219905 | 220385 |  | |  |  |  |  |  |  |  |  |  |  |  |  |  |
| MS14413_00325 | *yigZ*; translational initiation | 345378 | 345436 |  | |  |  |  |  |  |  |  |  |  |  |  |  |  |
| MS14413_00375 | *fabH;* beta-ketoacyl-ACP synthase III | 388376 | 388431 |  | |  |  |  |  |  |  |  |  |  |  |  |  |  |
| MS14413_00417 | *cybC;* cytochrome b | 433554 | 433554 |  | |  |  |  |  |  |  |  |  |  |  |  |  |  |
| Intergenic | - | 448420 | 448428 |  | |  |  |  |  |  |  |  |  |  |  |  |  |  |
| MS14413_00568 | histidine kinase | 586947 | 587007 |  | |  |  |  |  |  |  |  |  |  |  |  |  |  |
| MS14413_00715 | choline transporter | 727272 | 727326 |  | |  |  |  |  |  |  |  |  |  |  |  |  |  |
| MS14413_00756 | *pstC*; phosphate ABC transporter permease | 774883 | 774934 |  | |  |  |  |  |  |  |  |  |  |  |  |  |  |
| MS14413_00762 | 2,4-diaminobutyrate decarboxylase | 783104 | 783163 |  | |  |  |  |  |  |  |  |  |  |  |  |  |  |
| MS14413_00795 | membrane protein | 823712 | 823777 |  | |  |  |  |  |  |  |  |  |  |  |  |  |  |
| MS14413_00835 | L-aspartate oxidase | 863086 | 863163 |  | |  |  |  |  |  |  |  |  |  |  |  |  |  |
| Intergenic | - | 869489 | 869557 |  | |  |  |  |  |  |  |  |  |  |  |  |  |  |
| MS14413_00893 | transporter | 921026 | 921077 |  | |  |  |  |  |  |  |  |  |  |  |  |  |  |
| MS14413_00936 | glycerophosphoryl diester phosphodiesterase | 973607 | 973655 |  | |  |  |  |  |  |  |  |  |  |  |  |  |  |
| MS14413_01029 | *eptA;* lipid A phosphoethanolamine transferase | 1099361 | 1099361 |  | |  |  |  |  |  |  |  |  |  |  |  |  |  |
| MS14413_01033 | esterase | 1105252 | 1105309 |  | |  |  |  |  |  |  |  |  |  |  |  |  |  |
| Intergenic | - | 1144429 | 1144487 |  | |  |  |  |  |  |  |  |  |  |  |  |  |  |
| MS14413_01170 | acyl-CoA dehydrogenase | 1245784 | 1245833 |  | |  |  |  |  |  |  |  |  |  |  |  |  |  |
| MS14413_01201 | *secF*; preprotein translocase subunit SecF | 1279764 | 1279817 |  | |  |  |  |  |  |  |  |  |  |  |  |  |  |
| Intergenic | - | 1354490 | 1354547 |  | |  |  |  |  |  |  |  |  |  |  |  |  |  |
| MS14413_01421 | guanosine polyphosphate pyrophosphohydrolase/synthetase | 1486382 | 1486399 |  | |  |  |  |  |  |  |  |  |  |  |  |  |  |
| MS14413_01492 | transporter | 1561154 | 1561217 |  | |  |  |  |  |  |  |  |  |  |  |  |  |  |
| MS14413_01521 | MFS transporter | 1585414 | 1585476 |  | |  |  |  |  |  |  |  |  |  |  |  |  |  |
| MS14413_01569 | acetyl-coenzyme A synthetase | 1636208 | 1636275 |  | |  |  |  |  |  |  |  |  |  |  |  |  |  |
| MS14413_01572 | 16S rRNA (uracil(1498)-N(3))-methyltransferase | 1640258 | 1640326 |  | |  |  |  |  |  |  |  |  |  |  |  |  |  |
| MS14413_01576 | hypothetical protein | 1643722 | 1643775 |  | |  |  |  |  |  |  |  |  |  |  |  |  |  |
| MS14413_01578 | membrane protein | 1645520 | 1645581 |  | |  |  |  |  |  |  |  |  |  |  |  |  |  |
| MS14413_01580 | *pdxH;* pyridoxine/pyridoxamine 5'-phosphate oxidase | 1648378 | 1648440 |  | |  |  |  |  |  |  |  |  |  |  |  |  |  |
| MS14413_01595 | *ftsQ;* cell division protein FtsQ | 1667117 | 1667169 |  | |  |  |  |  |  |  |  |  |  |  |  |  |  |
| MS14413_01608 | Thiol disulfide interchange protein | 1680793 | 1680851 |  | |  |  |  |  |  |  |  |  |  |  |  |  |  |
| MS14413_01620 | oxidoreductase | 1691435 | 1691488 |  | |  |  |  |  |  |  |  |  |  |  |  |  |  |
| MS14413_01621 | *parE;* DNA topoisomerase IV subunit B | 1692838 | 1692890 |  | |  |  |  |  |  |  |  |  |  |  |  |  |  |
| MS14413_01641 | acetamidase | 1718198 | 1718210 |  | |  |  |  |  |  |  |  |  |  |  |  |  |  |
| MS14413_01656 | *glmS;* glutamine--fructose-6-phosphate aminotransferase | 1733400 | 1733459 |  | |  |  |  |  |  |  |  |  |  |  |  |  |  |
| MS14413_01664 | urocanate hydratase | 1743084 | 1743128 |  | |  |  |  |  |  |  |  |  |  |  |  |  |  |
| MS14413_01678 | sodium transporter | 1759250 | 1759303 |  | |  |  |  |  |  |  |  |  |  |  |  |  |  |
| MS14413_01817 | tyrosine protein kinase | 1902188 | 1902190 |  | |  |  |  |  |  |  |  |  |  |  |  |  |  |
| MS14413_01846 | UDP-glucose 6-dehydrogenase | 1932400 | 1932470 |  | |  |  |  |  |  |  |  |  |  |  |  |  |  |
| MS14413_01847 | glucose-6-phosphate isomerase | 1934545 | 1934590 |  | |  |  |  |  |  |  |  |  |  |  |  |  |  |
| MS14413_01848 | UDP-glucose 4-epimerase | 1935852 | 1935905 |  | |  |  |  |  |  |  |  |  |  |  |  |  |  |
| MS14413_01850 | L-lactate permease | 1938323 | 1938451 |  | |  |  |  |  |  |  |  |  |  |  |  |  |  |
| Intergenic | - | 1952022 | 1952085 |  | |  |  |  |  |  |  |  |  |  |  |  |  |  |
| MS14413_01891 | polysaccharide biosynthesis protein | 1970769 | 1970814 |  | |  |  |  |  |  |  |  |  |  |  |  |  |  |
| MS14413_01893 | UDP-glucose 6-dehydrogenase | 1973776 | 1973846 |  | |  |  |  |  |  |  |  |  |  |  |  |  |  |
| MS14413_01894 | glucose-6-phosphate isomerase | 1975921 | 1975966 |  | |  |  |  |  |  |  |  |  |  |  |  |  |  |
| MS14413_01895 | UDP-glucose 4-epimerase | 1977228 | 1977281 |  | |  |  |  |  |  |  |  |  |  |  |  |  |  |
| MS14413_01897 | L-lactate permease | 1979699 | 1979827 |  | |  |  |  |  |  |  |  |  |  |  |  |  |  |
| Intergenic | - | 1993410 | 1993474 |  | |  |  |  |  |  |  |  |  |  |  |  |  |  |
| Intergenic | - | 2126897 | 2127167 |  | |  |  |  |  |  |  |  |  |  |  |  |  |  |
| Intergenic | - | 2142198 | 2142777 |  | |  |  |  |  |  |  |  |  |  |  |  |  |  |
| MS14413_02118 | general secretion pathway protein | 2203613 | 2203666 |  | |  |  |  |  |  |  |  |  |  |  |  |  |  |
| MS14413_02145 | *htpG*; molecular chaperone HtpG | 2230316 | 2230365 |  | |  |  |  |  |  |  |  |  |  |  |  |  |  |
| MS14413_02161 | acyl-CoA thioesterase | 2247958 | 2248012 |  | |  |  |  |  |  |  |  |  |  |  |  |  |  |
| MS14413_02162 | CDP-diacylglycerol--glycerol-3-phosphate 3-phosphatidyltransferase | 2248553 | 2248617 |  | |  |  |  |  |  |  |  |  |  |  |  |  |  |
| MS14413_02168 | *hylD;* secretion protein HlyD | 2254751 | 2254802 |  | |  |  |  |  |  |  |  |  |  |  |  |  |  |
| Intergenic | - | 2340557 | 2340605 |  | |  |  |  |  |  |  |  |  |  |  |  |  |  |
| MS14413_02323 | 2-isopropylmalate synthase | 2418224 | 2418282 |  | |  |  |  |  |  |  |  |  |  |  |  |  |  |
| MS14413_02343 | tail-specific protease | 2447503 | 2447576 |  | |  |  |  |  |  |  |  |  |  |  |  |  |  |
| Intergenic | - | 2616132 | 2616183 |  | |  |  |  |  |  |  |  |  |  |  |  |  |  |
| MS14413_02597 | hypothetical protein | 2722502 | 2722573 |  | |  |  |  |  |  |  |  |  |  |  |  |  |  |
| MS14413_02683 | *fstK;* cell division protein FstK | 2810313 | 2810372 |  | |  |  |  |  |  |  |  |  |  |  |  |  |  |
| MS14413_02694 | ATP-dependent DNA helicase Rep | 2820563 | 2820620 |  | |  |  |  |  |  |  |  |  |  |  |  |  |  |
| MS14413_02706 | *pilT*; twitching motility protein PilT | 2831926 | 2831988 |  | |  |  |  |  |  |  |  |  |  |  |  |  |  |
| MS14413_02721 | integrase | 2851265 | 2851319 |  | |  |  |  |  |  |  |  |  |  |  |  |  |  |
| Intergenic | - | 2857979 | 2858042 |  | |  |  |  |  |  |  |  |  |  |  |  |  |  |
| MS14413_02740 | peptidase S24 | 2861126 | 2861197 |  | |  |  |  |  |  |  |  |  |  |  |  |  |  |
| MS14413_02777 | hypothetical protein | 2890668 | 2890728 |  | |  |  |  |  |  |  |  |  |  |  |  |  |  |
| Intergenic | - | 2897332 | 2897391 |  | |  |  |  |  |  |  |  |  |  |  |  |  |  |
| MS14413_02801 | type II methyltransferase | 2919692 | 2919752 |  | |  |  |  |  |  |  |  |  |  |  |  |  |  |
| MS14413_02823 | membrane protein | 2942416 | 2942464 |  | |  |  |  |  |  |  |  |  |  |  |  |  |  |
| MS14413_02829 | protein involved in heme utilization | 2950550 | 2950621 |  | |  |  |  |  |  |  |  |  |  |  |  |  |  |
| MS14413_02878 | *araC;* family transcriptional regulator AraC | 3001438 | 3001497 |  | |  |  |  |  |  |  |  |  |  |  |  |  |  |
| Intergenic | - | 3141318 | 3141318 |  | |  |  |  |  |  |  |  |  |  |  |  |  |  |
| Intergenic | - | 3177355 | 3177355 |  | |  |  |  |  |  |  |  |  |  |  |  |  |  |
| MS14413_03084 | Xaa-Pro aminopeptidase | 3180278 | 3180350 |  | |  |  |  |  |  |  |  |  |  |  |  |  |  |
| MS14413_03109 | dihydroorotase | 3208124 | 3208185 |  | |  |  |  |  |  |  |  |  |  |  |  |  |  |
| MS14413_03115 | *yigN*; Inner membrane protein YjgN | 3215478 | 3215495 |  | |  |  |  |  |  |  |  |  |  |  |  |  |  |
| MS14413_03166 | replicative DNA helicase | 3270658 | 3270712 |  | |  |  |  |  |  |  |  |  |  |  |  |  |  |
| MS14413_03170 | *cyoE;* protoheme IX farnesyltransferase | 3274064 | 3274559 |  | |  |  |  |  |  |  |  |  |  |  |  |  |  |
| MS14413_03173 | *cydA;* cytochrome ubiquinol oxidase subunit I | 3275751 | 3275819 |  | |  |  |  |  |  |  |  |  |  |  |  |  |  |
| MS14413_03178 | *pgaA*; poly-beta-1,6 N-acetyl-D-glucosamine export porin PgaA | 3285340 | 3285403 |  | |  |  |  |  |  |  |  |  |  |  |  |  |  |
| MS14413_03195 | AMP-binding protein | 3303102 | 3303153 |  | |  |  |  |  |  |  |  |  |  |  |  |  |  |
| MS14413_03317 | N-6 DNA methylase | 3434996 | 3435010 |  | |  |  |  |  |  |  |  |  |  |  |  |  |  |
| MS14413_03331 | hypothetical protein | 3442954 | 3443116 |  | |  |  |  |  |  |  |  |  |  |  |  |  |  |
| Intergenic | - | 3444464 | 3445295 |  | |  |  |  |  |  |  |  |  |  |  |  |  |  |
| MS14413_03346 | stress-responsive nuclear envelope protein | 3453748 | 3453990 |  | |  |  |  |  |  |  |  |  |  |  |  |  |  |
| MS14413_03349 | hypothetical protein | 3454769 | 3454959 |  | |  |  |  |  |  |  |  |  |  |  |  |  |  |
| MS14413_03351 | prophage PSSB64-0 | 3455730 | 3455797 |  | |  |  |  |  |  |  |  |  |  |  |  |  |  |
| Intergenic | - | 3459050 | 3459192 |  | |  |  |  |  |  |  |  |  |  |  |  |  |  |
| MS14413_03362 | phage tail tape measure protein | 3465326 | 3465326 |  | |  |  |  |  |  |  |  |  |  |  |  |  |  |
| MS14413_03366 | bacteriophage protein | 3470467 | 3470614 |  | |  |  |  |  |  |  |  |  |  |  |  |  |  |
| MS14413_03367 | hypothetical protein | 3471599 | 3472003 |  | |  |  |  |  |  |  |  |  |  |  |  |  |  |
| MS14413_03413 | *adeN*; TetR family transcriptional regulator | 3515298 | 3515507 |  | |  |  |  |  |  |  |  |  |  |  |  |  |  |
| MS14413_03521 | 4-hydroxybenzoate transporter | 3622246 | 3622302 |  | |  |  |  |  |  |  |  |  |  |  |  |  |  |
| MS14413_03553 | *tynA*; tyramine oxidase | 3653658 | 3653720 |  | |  |  |  |  |  |  |  |  |  |  |  |  |  |
| MS14413_03570 | 2,5-dioxovalerate dehydrogenase | 3674960 | 3674960 |  | |  |  |  |  |  |  |  |  |  |  |  |  |  |
| MS14413_03603 | transposase | 3710975 | 3710975 |  | |  |  |  |  |  |  |  |  |  |  |  |  |  |
| MS14413_03609 | transposase | 3724070 | 3724070 |  | |  |  |  |  |  |  |  |  |  |  |  |  |  |
| MS14413_03632 | 2,5-dioxovalerate dehydrogenase | 3749705 | 3749705 |  | |  |  |  |  |  |  |  |  |  |  |  |  |  |
| MS14413_03689 | *adeS*; two-component sensor histidine kinase | 3804535 | 3804546 |  | |  |  |  |  |  |  |  |  |  |  |  |  |  |
| Intergenic | - | 3821781 | 3821781 |  | |  |  |  |  |  |  |  |  |  |  |  |  |  |
| MS14413_03759 | TonB-dependent receptor | 3884025 | 3884083 |  | |  |  |  |  |  |  |  |  |  |  |  |  |  |
| MS14413_03883 | heme-binding protein | 4013662 | 4013733 |  | |  |  |  |  |  |  |  |  |  |  |  |  |  |
| MS14413_03912 | deoxyribonuclease | 4041162 | 4041233 |  | |  |  |  |  |  |  |  |  |  |  |  |  |  |
| MS14413_03925 | *lptD*; LPS-assembly protein LptD | 4054214 | 4054281 |  | |  |  |  |  |  |  |  |  |  |  |  |  |  |
