# Supplementary material for: Rescuing Tetracycline Class Antibiotics for the Treatment of Multidrug-Resistant Acinetobacter baumannii Pulmonary Infection
Source: mBio. 2022 Jan 11;13(1):e03517-21. doi: 10.1128/mbio.03517-21 (PMC8749419; doi:10.1128/mbio.03517-21)
Supplement: TABLE S2 [file mbio.03517-21-st002.docx]

**TABLE S2A** *A. baumannii* MS14413 up-regulated genes following 1 h treatment with 64 μM PBT2 in CA-MHB grown at 37°C.

| **Locus_tag** | **LogFC** | **FDR** | **Gene** | **Product** |
| --- | --- | --- | --- | --- |
| MS14413_RS07940 | 6.11 | 1.11E-09 | *cntO* | TonB-dependent receptor |
| MS14413_RS02630 | 5.67 | 1.15E-08 | *adeF* | multidrug efflux RND transporter periplasmic adaptor subunit AdeF |
| MS14413_RS07060 | 5.57 | 2.71E-11 | *-* | (2Fe-2S)-binding protein |
| MS14413_RS19085 | 5.46 | 9.27E-10 | *-* | siderophore biosynthesis protein |
| MS14413_RS19080 | 4.94 | 1.83E-09 | *-* | SidA/IucD/PvdA family monooxygenase |
| MS14413_RS15415 | 4.66 | 9.03E-10 | *cntO* | TonB-dependent receptor |
| MS14413_RS15330 | 4.62 | 9.25E-13 | *czcE* | cation diffusion facilitator transporter |
| MS14413_RS18965 | 4.51 | 1.34E-09 | *fpvA* | TonB-dependent siderophore receptor |
| MS14413_RS02635 | 4.42 | 1.54E-09 | *adeG* | multidrug efflux RND transporter permease subunit AdeG |
| MS14413_RS04145 | 4.33 | 4.61E-10 | *-* | sulfate ABC transporter substrate-binding protein |
| MS14413_RS15815 | 4.16 | 2.38E-10 | *-* | hypothetical protein |
| MS14413_RS14360 | 4.15 | 4.54E-10 | *pfeA* | TonB-dependent siderophore receptor |
| MS14413_RS19075 | 4.02 | 9.86E-09 | *-* | DHA2 family efflux MFS transporter permease subunit |
| MS14413_RS18485 | 3.96 | 1.02E-08 | *-* | transcriptional repressor |
| MS14413_RS18015 | 3.95 | 1.50E-10 | *-* | MFS transporter |
| MS14413_RS06785 | 3.95 | 1.59E-09 | *-* | EamA family transporter |
| MS14413_RS04150 | 3.93 | 4.63E-09 | *-* | alpha/beta hydrolase |
| MS14413_RS00640 | 3.90 | 1.33E-08 | *-* | amino acid ABC transporter permease |
| MS14413_RS07625 | 3.89 | 3.32E-09 | *-* | AzlD domain-containing protein |
| MS14413_RS01755 | 3.86 | 7.41E-07 | *-* | transposase |
| MS14413_RS00375 | 3.86 | 7.22E-09 | *arsC* | arsenate reductase (glutaredoxin) |
| MS14413_RS00645 | 3.79 | 4.28E-09 | *-* | amino acid ABC transporter permease |
| MS14413_RS01730 | 3.76 | 2.55E-09 | *-* | cold-shock protein |
| MS14413_RS05345 | 3.69 | 1.19E-11 | *-* | PepSY domain-containing protein |
| MS14413_RS08905 | 3.68 | 1.38E-08 | *-* | sulfonate ABC transporter substrate-binding protein |
| MS14413_RS02750 | 3.65 | 2.89E-08 | *-* | OmpW family protein |
| MS14413_RS02495 | 3.62 | 1.19E-11 | *-* | alpha/beta hydrolase |
| MS14413_RS11500 | 3.48 | 4.79E-10 | *-* | TonB-dependent siderophore receptor |
| MS14413_RS00195 | 3.47 | 3.43E-08 | *-* | SfnB family sulfur acquisition oxidoreductase |
| MS14413_RS03395 | 3.43 | 9.57E-07 | *bauD* | ferric acinetobactin ABC transporter permease subunit BauD |
| MS14413_RS18970 | 3.43 | 2.44E-07 | *-* | hypothetical protein |
| MS14413_RS00635 | 3.41 | 1.07E-07 | *-* | amino acid ABC transporter ATP-binding protein |
| MS14413_RS16225 | 3.33 | 1.16E-07 | *-* | TonB-dependent siderophore receptor |
| MS14413_RS07665 | 3.31 | 8.27E-10 | *-* | TetR/AcrR family transcriptional regulator |
| MS14413_RS08900 | 3.30 | 3.02E-07 | *-* | sulfonate ABC transporter substrate-binding protein |
| MS14413_RS04155 | 3.28 | 1.15E-06 | *cysT* | sulfate ABC transporter permease subunit CysT |
| MS14413_RS03440 | 3.27 | 2.30E-08 | *-* | TetR/AcrR family transcriptional regulator |
| MS14413_RS03400 | 3.26 | 6.63E-08 | *basB* | acinetobactin non-ribosomal peptide synthetase subunit BasB |
| MS14413_RS03360 | 3.26 | 2.01E-08 | *basE* | (2,2C3-dihydroxybenzoyl)adenylate synthase BasE |
| MS14413_RS03410 | 3.25 | 7.37E-10 | *bauF* | acinetobactin utilization protein BauF |
| MS14413_RS18975 | 3.23 | 4.56E-08 | *-* | PepSY domain-containing protein |
| MS14413_RS00430 | 3.22 | 3.99E-09 | *tauA* | taurine ABC transporter substrate-binding protein |
| MS14413_RS15810 | 3.22 | 9.45E-12 | *-* | LLM class flavin-dependent oxidoreductase |
| MS14413_RS00365 | 3.18 | 5.31E-08 | *arsB* | ACR3 family arsenite efflux transporter |
| MS14413_RS17850 | 3.17 | 2.61E-09 | *-* | TetR/AcrR family transcriptional regulator |
| MS14413_RS03390 | 3.14 | 1.35E-06 | *bauC* | ferric acinetobactin ABC transporter permease subunit BauC |
| MS14413_RS07620 | 3.12 | 5.66E-09 | *-* | AzlC family ABC transporter permease |
| MS14413_RS18310 | 3.04 | 6.10E-09 | *adeA* | multidrug efflux RND transporter periplasmic adaptor subunit AdeA |
| MS14413_RS07655 | 3.03 | 5.17E-07 | *-* | DUF1304 domain-containing protein |
| MS14413_RS04320 | 3.02 | 6.24E-09 | *-* | CSLREA domain-containing protein |
| MS14413_RS07085 | 3.01 | 2.87E-07 | *-* | pilin |
| MS14413_RS07500 | 3.00 | 2.55E-08 | *-* | helix-turn-helix transcriptional regulator |
| MS14413_RS18650 | 2.98 | 2.48E-09 | *-* | MFS transporter |
| MS14413_RS04160 | 2.96 | 8.18E-06 | *cysW* | sulfate ABC transporter permease subunit CysW |
| MS14413_RS17845 | 2.94 | 1.39E-08 | *-* | MBL fold metallo-hydrolase |
| MS14413_RS03355 | 2.94 | 3.64E-08 | *basF* | acinetobactin biosynthesis bifunctional isochorismatase/aryl carrier protein BasF |
| MS14413_RS10340 | 2.91 | 6.71E-11 | *feoA* | ferrous iron transport protein A |
| MS14413_RS01225 | 2.89 | 7.10E-07 | *-* | hypothetical protein |
| MS14413_RS05690 | 2.89 | 9.27E-10 | *-* | peroxiredoxin |
| MS14413_RS07660 | 2.83 | 1.15E-08 | *-* | zinc-binding dehydrogenase |
| MS14413_RS00765 | 2.83 | 1.37E-07 | *-* | hypothetical protein |
| MS14413_RS07860 | 2.79 | 9.45E-12 | *-* | TonB-dependent siderophore receptor |
| MS14413_RS11385 | 2.78 | 5.66E-09 | *-* | energy transducer TonB |
| MS14413_RS02640 | 2.78 | 9.53E-08 | *adeH* | multidrug efflux RND transporter outer membrane subunit AdeH |
| MS14413_RS10195 | 2.76 | 2.69E-09 | *-* | IS*91*-like element IS*Vsa3* family transposase |
| MS14413_RS17305 | 2.67 | 2.18E-09 | *-* | TonB-dependent siderophore receptor |
| MS14413_RS07650 | 2.65 | 4.17E-07 | *-* | GlpM family protein |
| MS14413_RS00200 | 2.64 | 3.10E-07 | *-* | SfnB family sulfur acquisition oxidoreductase |
| MS14413_RS18495 | 2.63 | 2.48E-09 | *-* | SDR family oxidoreductase |
| MS14413_RS18950 | 2.62 | 7.64E-08 | *-* | HlyD family efflux transporter periplasmic adaptor subunit |
| MS14413_RS18010 | 2.61 | 2.89E-09 | *-* | HlyD family secretion protein |
| MS14413_RS03365 | 2.59 | 3.65E-08 | *basD* | acinetobactin non-ribosomal peptide synthetase subunit BasD |
| MS14413_RS00420 | 2.59 | 1.21E-07 | *tauC* | taurine ABC transporter permease TauC |
| MS14413_RS19050 | 2.58 | 1.33E-08 | *-* | TonB-dependent receptor |
| MS14413_RS00425 | 2.57 | 6.25E-08 | *-* | ATP-binding cassette domain-containing protein |
| MS14413_RS08895 | 2.56 | 3.99E-06 | *ssuD* | FMNH2-dependent alkanesulfonate monooxygenase |
| MS14413_RS15965 | 2.56 | 1.80E-06 | *-* | hypothetical protein |
| MS14413_RS13485 | 2.52 | 1.21E-08 | *ggt* | gamma-glutamyltransferase |
| MS14413_RS11185 | 2.52 | 1.41E-07 | *-* | hypothetical protein |
| MS14413_RS00630 | 2.52 | 1.32E-06 | *-* | transporter substrate-binding domain-containing protein |
| MS14413_RS10200 | 2.52 | 1.83E-10 | *-* | hypothetical protein |
| MS14413_RS03775 | 2.52 | 1.04E-09 | *-* | ferredoxin reductase |
| MS14413_RS16185 | 2.51 | 1.61E-09 | *-* | TonB-dependent receptor |
| MS14413_RS00140 | 2.51 | 1.24E-08 | *-* | hypothetical protein |
| MS14413_RS18945 | 2.49 | 1.62E-06 | *-* | ABC transporter ATP-binding protein |
| MS14413_RS00115 | 2.49 | 3.88E-07 | *-* | cytosine permease |
| MS14413_RS02540 | 2.49 | 1.83E-09 | *-* | hemin uptake protein HemP |
| MS14413_RS18980 | 2.47 | 6.32E-07 | *-* | hypothetical protein |
| MS14413_RS19070 | 2.43 | 2.52E-08 | *acsC* | siderophore achromobactin biosynthesis protein AcsC |
| MS14413_RS14015 | 2.38 | 1.89E-08 | *-* | sigma-70 family RNA polymerase sigma factor |
| MS14413_RS18785 | 2.37 | 1.51E-08 | *-* | transcriptional regulator |
| MS14413_RS08250 | 2.37 | 1.09E-07 | *hutH* | histidine ammonia-lyase |
| MS14413_RS19045 | 2.37 | 8.74E-07 | *-* | hypothetical protein |
| MS14413_RS03385 | 2.34 | 2.61E-06 | *bauE* | ferric acinetobactin ABC transporter ATP-binding protein BauE |
| MS14413_RS06860 | 2.34 | 7.45E-10 | *-* | hypothetical protein |
| MS14413_RS17450 | 2.33 | 2.02E-07 | *-* | 3-oxoacid CoA-transferase subunit A |
| MS14413_RS10345 | 2.32 | 8.19E-10 | *feoB* | ferrous iron transporter B |
| MS14413_RS11120 | 2.30 | 1.68E-09 | *-* | Rne/Rng family ribonuclease |
| MS14413_RS17940 | 2.30 | 5.70E-07 | *-* | SLC13 family permease |
| MS14413_RS01725 | 2.29 | 2.44E-06 | *-* | hypothetical protein |
| MS14413_RS14035 | 2.29 | 8.03E-09 | *-* | DUF560 domain-containing protein |
| MS14413_RS00370 | 2.27 | 4.27E-07 | *-* | metalloregulator ArsR/SmtB family transcription factor |
| MS14413_RS12580 | 2.27 | 2.56E-06 | *-* | hypothetical protein |
| MS14413_RS19265 | 2.26 | 4.27E-08 | *-* | ABC transporter substrate-binding protein |
| MS14413_RS09975 | 2.24 | 3.79E-09 | *-* | DUF2726 domain-containing protein |
| MS14413_RS19065 | 2.24 | 3.31E-08 | *-* | IucA/IucC family siderophore biosynthesis protein |
| MS14413_RS12450 | 2.24 | 1.65E-06 | *-* | MFS transporter |
| MS14413_RS03350 | 2.21 | 4.48E-07 | *basG* | acinetobactin biosynthesis histidine decarboxylase BasG |
| MS14413_RS08980 | 2.21 | 1.82E-07 | *-* | hypothetical protein |
| MS14413_RS08890 | 2.18 | 5.79E-05 | *ssuC* | aliphatic sulfonate ABC transporter permease SsuC |
| MS14413_RS01150 | 2.16 | 4.59E-09 | *-* | L-serine ammonia-lyase |
| MS14413_RS18045 | 2.15 | 5.55E-07 | *-* | NAD-dependent epimerase/dehydratase family protein |
| MS14413_RS02880 | 2.15 | 3.50E-07 | *-* | peptide MFS transporter |
| MS14413_RS01710 | 2.13 | 1.83E-09 | *-* | hypothetical protein |
| MS14413_RS07735 | 2.12 | 1.05E-08 | *-* | cation acetate symporter |
| MS14413_RS17505 | 2.12 | 5.02E-09 | *-* | carbohydrate porin |
| MS14413_RS07470 | 2.12 | 1.60E-08 | *-* | hypothetical protein |
| MS14413_RS18490 | 2.11 | 1.61E-06 | *-* | isochorismatase family protein |
| MS14413_RS00790 | 2.11 | 8.10E-08 | *-* | methylcrotonoyl-CoA carboxylase |
| MS14413_RS00205 | 2.11 | 8.59E-06 | *-* | LLM class flavin-dependent oxidoreductase |
| MS14413_RS06965 | 2.10 | 9.36E-07 | *-* | lipase secretion chaperone |
| MS14413_RS12500 | 2.10 | 1.85E-07 | *-* | AraC family transcriptional regulator |
| MS14413_RS18190 | 2.10 | 4.72E-08 | *iaaH* | indoleacetamide hydrolase |
| MS14413_RS18110 | 2.09 | 8.69E-10 | *-* | ribose-phosphate pyrophosphokinase |
| MS14413_RS08245 | 2.09 | 5.94E-07 | *-* | amino acid permease |
| MS14413_RS18775 | 2.09 | 9.58E-08 | *-* | thiamine pyrophosphate-dependent dehydrogenase E1 component subunit alpha |
| MS14413_RS06170 | 2.08 | 2.29E-05 | *-* | acyl-CoA dehydrogenase family protein |
| MS14413_RS09570 | 2.08 | 1.71E-08 | *-* | AMP-binding protein |
| MS14413_RS18150 | 2.08 | 4.35E-05 | *-* | hypothetical protein |
| MS14413_RS17755 | 2.07 | 1.15E-06 | *-* | DUF485 domain-containing protein |
| MS14413_RS14020 | 2.06 | 1.03E-07 | *-* | FecR domain-containing protein |
| MS14413_RS18185 | 2.06 | 2.96E-07 | *-* | LuxR family transcriptional regulator |
| MS14413_RS00785 | 2.06 | 4.06E-09 | *-* | isovaleryl-CoA dehydrogenase |
| MS14413_RS18940 | 2.06 | 6.20E-06 | *-* | ABC transporter permease |
| MS14413_RS06690 | 2.05 | 1.80E-06 | *-* | DEAD/DEAH box helicase |
| MS14413_RS00415 | 2.04 | 1.26E-06 | *tauD* | taurine dioxygenase |
| MS14413_RS13960 | 2.04 | 1.57E-06 | *-* | hypothetical protein |
| MS14413_RS18700 | 2.04 | 5.91E-09 | *-* | NAD(P)H-dependent oxidoreductase |
| MS14413_RS15985 | 2.03 | 2.19E-08 | *-* | DUF4198 domain-containing protein |
| MS14413_RS10190 | 2.02 | 1.42E-06 | *-* | hypothetical protein |
| MS14413_RS07785 | 2.02 | 1.63E-09 | *acs* | acetate--CoA ligase |
| MS14413_RS00625 | 2.02 | 8.12E-06 | *-* | cysteine ABC transporter substrate-binding protein |
| MS14413_RS00450 | 2.02 | 2.01E-08 | *-* | monooxygenase |

**TABLE S2B** *A. baumannii* MS14413 down-regulated genes following 1 h treatment with 64 μM PBT2 in CA-MHB grown at 37°C.

| **Locus_tag** | **LogFC** | **FDR** | **Gene** | **Product** |
| --- | --- | --- | --- | --- |
| MS14413_RS17135 | -2.01 | 1.17E-07 | *-* | hypothetical protein |
| MS14413_RS10595 | -2.01 | 3.60E-09 | *-* | DUF1311 domain-containing protein |
| MS14413_RS00675 | -2.02 | 4.61E-10 | *-* | Lrp/AsnC family transcriptional regulator |
| MS14413_RS12360 | -2.03 | 1.83E-10 | *rpe* | ribulose-phosphate 3-epimerase |
| MS14413_RS02160 | -2.03 | 2.01E-06 | *-* | hypothetical protein |
| MS14413_RS02330 | -2.04 | 1.38E-10 | *-* | ATP-binding protein |
| MS14413_RS04920 | -2.05 | 5.60E-10 | *-* | hypothetical protein |
| MS14413_RS18510 | -2.05 | 2.20E-10 | *modA* | molybdate ABC transporter substrate-binding protein |
| MS14413_RS03475 | -2.05 | 4.63E-09 | *yddG* | aromatic amino acid DMT transporter YddG |
| MS14413_RS09855 | -2.06 | 4.61E-10 | *-* | TetR/AcrR family transcriptional regulator |
| MS14413_RS02895 | -2.07 | 6.86E-08 | *-* | hypothetical protein |
| MS14413_RS18730 | -2.07 | 1.70E-06 | *-* | TIGR01244 family phosphatase |
| MS14413_RS13470 | -2.08 | 1.28E-10 | *-* | thioesterase family protein |
| MS14413_RS01985 | -2.09 | 1.58E-10 | *crp* | cAMP-activated global transcriptional regulator CRP |
| MS14413_RS00075 | -2.10 | 2.48E-09 | *-* | type 1 fimbrial protein |
| MS14413_RS02890 | -2.12 | 5.60E-10 | *-* | tyrosine-type recombinase/integrase |
| MS14413_RS09900 | -2.13 | 2.32E-11 | *-* | DUF2236 domain-containing protein |
| MS14413_RS01605 | -2.13 | 2.13E-10 | *-* | O-methyltransferase |
| MS14413_RS17390 | -2.19 | 1.09E-09 | *-* | NADPH-dependent 2,2C4-dienoyl-CoA reductase |
| MS14413_RS10580 | -2.21 | 2.32E-11 | *-* | alpha/beta fold hydrolase |
| MS14413_RS18735 | -2.21 | 1.23E-06 | *-* | MBL fold metallo-hydrolase |
| MS14413_RS08290 | -2.23 | 4.71E-11 | *fahA* | fumarylacetoacetase |
| MS14413_RS15445 | -2.24 | 4.81E-11 | *-* | NADPH-dependent 2,2C4-dienoyl-CoA reductase |
| MS14413_RS10665 | -2.25 | 2.60E-09 | *-* | peptidoglycan hydrolase |
| MS14413_RS19105 | -2.26 | 2.13E-10 | *-* | acyl-CoA dehydrogenase family protein |
| MS14413_RS02225 | -2.26 | 6.78E-10 | *-* | alpha/beta fold hydrolase |
| MS14413_RS10590 | -2.27 | 4.63E-06 | *-* | outer membrane beta-barrel protein |
| MS14413_RS02600 | -2.40 | 3.92E-09 | *-* | multidrug efflux SMR transporter AbeS |
| MS14413_RS16285 | -2.46 | 2.87E-11 | *benE* | benzoate/H(+) symporter BenE family transporter |
| MS14413_RS18635 | -2.46 | 8.71E-09 | *-* | hypothetical protein |
| MS14413_RS04220 | -2.53 | 5.63E-11 | *-* | enoyl-CoA hydratase/isomerase family protein |
| MS14413_RS16015 | -2.54 | 4.83E-10 | *-* | type II toxin-antitoxin system RelB/DinJ family antitoxin |
| MS14413_RS16315 | -2.55 | 8.90E-11 | *-* | acetyl-CoA C-acetyltransferase |
| MS14413_RS10970 | -2.61 | 1.17E-09 | *-* | hypothetical protein |
| MS14413_RS11970 | -2.62 | 1.99E-11 | *-* | crotonase/enoyl-CoA hydratase family protein |
| MS14413_RS13390 | -2.63 | 4.02E-07 | *-* | bacteriohemerythrin |
| MS14413_RS08765 | -2.71 | 2.60E-09 | *-* | matrixin family metalloprotease |
| MS14413_RS16740 | -2.75 | 2.89E-09 | *-* | Arc family DNA-binding protein |
| MS14413_RS02595 | -2.85 | 7.49E-08 | *-* | YfhL family 4Fe-4S dicluster ferredoxin |
| MS14413_RS11850 | -2.99 | 2.32E-11 | *-* | TetR/AcrR family transcriptional regulator |
| MS14413_RS02590 | -3.08 | 2.01E-08 | *-* | tRNA 5-hydroxyuridine modification protein YegQ |
| MS14413_RS10975 | -3.17 | 1.42E-11 | *-* | tRNA-binding protein |
| MS14413_RS16915 | -3.23 | 2.38E-10 | *-* | hypothetical protein |
| MS14413_RS00680 | -3.88 | 4.48E-12 | *-* | LysE family translocator |
| MS14413_RS15335 | -4.60 | 4.95E-13 | *-* | amino acid transporter |

**TABLE S2C** *A. baumannii* MS14413 up-regulated genes following 1 h treatment with 64 μM PBT2 and 8 μM ZnSO_4_ in CA-MHB grown at 37°C.

| **Locus_tag** | **LogFC** | **FDR** | **Gene** | **Product** |
| --- | --- | --- | --- | --- |
| MS14413_RS02630 | 6.70 | 2.37E-09 | *adeF* | multidrug efflux RND transporter periplasmic adaptor subunit AdeF |
| MS14413_RS02635 | 5.85 | 1.49E-10 | *adeG* | multidrug efflux RND transporter permease subunit AdeG |
| MS14413_RS07940 | 5.02 | 3.80E-09 | *cntO* | TonB-dependent receptor |
| MS14413_RS19085 | 4.84 | 2.30E-09 | *-* | siderophore biosynthesis protein |
| MS14413_RS07060 | 4.80 | 1.23E-10 | *-* | (2Fe-2S)-binding protein |
| MS14413_RS15330 | 4.67 | 6.97E-13 | *czcE* | cation diffusion facilitator protein |
| MS14413_RS19080 | 4.59 | 3.16E-09 | *-* | SidA/IucD/PvdA family monooxygenase |
| MS14413_RS15815 | 4.56 | 1.16E-10 | *-* | hypothetical protein |
| MS14413_RS02640 | 4.54 | 7.90E-10 | *adeH* | multidrug efflux RND transporter outer membrane subunit AdeH |
| MS14413_RS19075 | 4.25 | 6.01E-09 | *-* | DHA2 family efflux MFS transporter permease subunit |
| MS14413_RS15810 | 3.94 | 1.29E-12 | *-* | LLM class flavin-dependent oxidoreductase |
| MS14413_RS15415 | 3.53 | 7.34E-09 | *cntO* | TonB-dependent receptor |
| MS14413_RS06785 | 3.50 | 4.81E-09 | *-* | EamA family transporter |
| MS14413_RS19065 | 3.40 | 6.23E-10 | *-* | IucA/IucC family siderophore biosynthesis protein |
| MS14413_RS18965 | 3.24 | 2.43E-08 | *fpvA* | TonB-dependent siderophore receptor |
| MS14413_RS10340 | 3.23 | 2.60E-11 | *feoA* | ferrous iron transport protein A |
| MS14413_RS01730 | 3.22 | 9.28E-09 | *-* | cold-shock protein |
| MS14413_RS18310 | 3.21 | 3.27E-09 | *adeA* | multidrug efflux RND transporter periplasmic adaptor subunit AdeA |
| MS14413_RS07665 | 3.20 | 8.16E-10 | *-* | TetR/AcrR family transcriptional regulator |
| MS14413_RS18015 | 3.20 | 8.47E-10 | *-* | MFS transporter |
| MS14413_RS19070 | 3.16 | 2.24E-09 | *acsC* | siderophore achromobactin biosynthesis protein AcsC |
| MS14413_RS02495 | 3.15 | 4.43E-11 | *-* | alpha/beta hydrolase |
| MS14413_RS07500 | 3.12 | 1.81E-08 | *-* | helix-turn-helix transcriptional regulator |
| MS14413_RS02750 | 3.10 | 1.28E-07 | *-* | OmpW family protein |
| MS14413_RS00430 | 3.06 | 5.16E-09 | *tauA* | taurine ABC transporter substrate-binding protein |
| MS14413_RS00375 | 3.00 | 9.77E-08 | *arsC* | arsenate reductase (glutaredoxin) |
| MS14413_RS01755 | 2.97 | 1.25E-05 | *-* | transposase |
| MS14413_RS18485 | 2.88 | 2.86E-07 | *-* | transcriptional repressor |
| MS14413_RS05345 | 2.87 | 7.15E-11 | *-* | PepSY domain-containing protein |
| MS14413_RS07625 | 2.83 | 8.59E-08 | *-* | AzlD domain-containing protein |
| MS14413_RS04145 | 2.79 | 1.89E-08 | *-* | sulfate ABC transporter substrate-binding protein |
| MS14413_RS14360 | 2.75 | 1.32E-08 | *pfeA* | TonB-dependent siderophore receptor |
| MS14413_RS11500 | 2.71 | 2.39E-09 | *-* | TonB-dependent siderophore receptor |
| MS14413_RS18950 | 2.68 | 6.49E-08 | *-* | HlyD family efflux transporter periplasmic adaptor subunit |
| MS14413_RS03400 | 2.63 | 6.58E-07 | *basB* | acinetobactin non-ribosomal peptide synthetase subunit BasB |
| MS14413_RS07470 | 2.59 | 2.54E-09 | *-* | hypothetical protein |
| MS14413_RS04320 | 2.57 | 2.65E-08 | *-* | CSLREA domain-containing protein |
| MS14413_RS19060 | 2.56 | 5.19E-09 | *-* | (2Fe-2S)-binding protein |
| MS14413_RS17305 | 2.56 | 2.82E-09 | *-* | TonB-dependent siderophore receptor |
| MS14413_RS03410 | 2.54 | 7.22E-09 | *bauF* | acinetobactin utilization protein BauF |
| MS14413_RS05690 | 2.54 | 2.42E-09 | *-* | peroxiredoxin |
| MS14413_RS00640 | 2.53 | 1.31E-06 | *-* | amino acid ABC transporter permease |
| MS14413_RS10195 | 2.50 | 5.80E-09 | *-* | IS*91*-like element IS*Vsa3* family transposase |
| MS14413_RS07620 | 2.49 | 4.39E-08 | *-* | AzlC family ABC transporter permease |
| MS14413_RS10345 | 2.49 | 3.34E-10 | *feoB* | ferrous iron transporter B |
| MS14413_RS00645 | 2.48 | 3.54E-07 | *-* | amino acid ABC transporter permease |
| MS14413_RS18650 | 2.45 | 1.66E-08 | *-* | MFS transporter |
| MS14413_RS17850 | 2.45 | 3.25E-08 | *-* | TetR/AcrR family transcriptional regulator |
| MS14413_RS10200 | 2.43 | 2.02E-10 | *-* | hypothetical protein |
| MS14413_RS01225 | 2.43 | 5.73E-06 | *-* | hypothetical protein |
| MS14413_RS11120 | 2.41 | 8.57E-10 | *-* | Rne/Rng family ribonuclease |
| MS14413_RS16225 | 2.39 | 4.11E-06 | *-* | TonB-dependent siderophore receptor |
| MS14413_RS18315 | 2.38 | 1.26E-08 | *adeB* | multidrug efflux RND transporter permease subunit AdeB |
| MS14413_RS15965 | 2.36 | 4.71E-06 | *-* | hypothetical protein |
| MS14413_RS07660 | 2.34 | 4.55E-08 | *-* | zinc-binding dehydrogenase |
| MS14413_RS18215 | 2.34 | 2.05E-07 | *-* | TolC family protein |
| MS14413_RS19050 | 2.32 | 2.76E-08 | *-* | TonB-dependent receptor |
| MS14413_RS06860 | 2.32 | 6.23E-10 | *-* | hypothetical protein |
| MS14413_RS07860 | 2.30 | 3.38E-11 | *-* | TonB-dependent siderophore receptor |
| MS14413_RS12500 | 2.28 | 8.37E-08 | *-* | AraC family transcriptional regulator |
| MS14413_RS17845 | 2.25 | 1.83E-07 | *-* | MBL fold metallo-hydrolase |
| MS14413_RS10190 | 2.24 | 5.52E-07 | *-* | hypothetical protein |
| MS14413_RS18320 | 2.24 | 1.36E-08 | *adeC* | multidrug efflux RND transporter outer membrane channel subunit AdeC |
| MS14413_RS17125 | 2.23 | 8.85E-08 | *-* | universal stress protein |
| MS14413_RS03775 | 2.21 | 2.24E-09 | *-* | ferredoxin reductase |
| MS14413_RS08245 | 2.19 | 3.73E-07 | *-* | amino acid permease |
| MS14413_RS07750 | 2.16 | 7.08E-09 | *-* | hypothetical protein |
| MS14413_RS00635 | 2.16 | 1.44E-05 | *-* | amino acid ABC transporter ATP-binding protein |
| MS14413_RS03355 | 2.16 | 9.91E-07 | *basF* | acinetobactin biosynthesis bifunctional isochorismatase/aryl carrier protein BasF |
| MS14413_RS01155 | 2.15 | 1.17E-08 | *-* | APC family permease |
| MS14413_RS18435 | 2.12 | 1.61E-07 | *-* | hypothetical protein |
| MS14413_RS18010 | 2.11 | 2.29E-08 | *-* | HlyD family secretion protein |
| MS14413_RS03390 | 2.11 | 9.69E-05 | *bauC* | ferric acinetobactin ABC transporter permease subunit BauC |
| MS14413_RS10205 | 2.09 | 7.55E-10 | *-* | aminoglycoside O-phosphotransferase APH(6)-Id |
| MS14413_RS03395 | 2.07 | 2.18E-04 | *bauD* | ferric acinetobactin ABC transporter permease subunit BauD |
| MS14413_RS18110 | 2.05 | 8.47E-10 | *-* | ribose-phosphate pyrophosphokinase |
| MS14413_RS18495 | 2.04 | 2.76E-08 | *-* | SDR family oxidoreductase |
| MS14413_RS18700 | 2.03 | 6.38E-09 | *-* | NAD(P)H-dependent oxidoreductase |
| MS14413_RS02425 | 2.03 | 5.19E-09 | *-* | cold-shock protein |
| MS14413_RS03360 | 2.03 | 3.02E-06 | *basE* | (2,2C3-dihydroxybenzoyl)adenylate synthase BasE |
| MS14413_RS18970 | 2.02 | 6.41E-05 | *-* | hypothetical protein |
| MS14413_RS18785 | 2.02 | 8.06E-08 | *-* | transcriptional regulator |
| MS14413_RS08905 | 2.02 | 7.69E-06 | *-* | sulfonate ABC transporter substrate-binding protein |
| MS14413_RS03440 | 2.02 | 3.83E-06 | *-* | TetR/AcrR family transcriptional regulator |
| MS14413_RS19260 | 2.02 | 3.21E-08 | *-* | Zn-dependent oligopeptidase |
| MS14413_RS04150 | 2.01 | 4.71E-06 | *-* | alpha/beta hydrolase |
| MS14413_RS04645 | 2.01 | 3.84E-09 | *-* | Ig-like domain repeat protein |
| MS14413_RS18975 | 2.00 | 7.43E-06 | *-* | PepSY domain-containing protein |
| MS14413_RS10210 | 2.00 | 8.16E-10 | *aph(3'')-Ib* | aminoglycoside O-phosphotransferase APH(3'')-Ib |

**TABLE S2D** *A. baumannii* MS14413 down-regulated genes following 1 h treatment with 64 μM PBT2 and 8 μM ZnSO_4_ in CA-MHB grown at 37°C.

| **Locus_tag** | **LogFC** | **FDR** | **Gene** | **Product** |
| --- | --- | --- | --- | --- |
| MS14413_RS05115 | -2.01 | 5.52E-10 | *-* | MFS transporter |
| MS14413_RS13235 | -2.01 | 9.41E-08 | *-* | hypothetical protein |
| MS14413_RS08915 | -2.02 | 3.82E-09 | *-* | RcnB family protein |
| MS14413_RS02895 | -2.02 | 1.15E-07 | *-* | hypothetical protein |
| MS14413_RS03470 | -2.03 | 2.10E-07 | *-* | DMT family transporter |
| MS14413_RS05800 | -2.03 | 2.54E-09 | *-* | acyl-CoA dehydrogenase C-terminal domain-containing protein |
| MS14413_RS09905 | -2.04 | 4.43E-07 | *-* | TetR/AcrR family transcriptional regulator |
| MS14413_RS10880 | -2.04 | 2.60E-11 | *-* | hypothetical protein |
| MS14413_RS07890 | -2.05 | 1.80E-10 | *-* | DUF721 domain-containing protein |
| MS14413_RS02980 | -2.06 | 8.61E-09 | *-* | hypothetical protein |
| MS14413_RS04960 | -2.07 | 1.27E-07 | *-* | helix-turn-helix transcriptional regulator |
| MS14413_RS12925 | -2.07 | 2.87E-09 | *-* | GntR family transcriptional regulator |
| MS14413_RS01435 | -2.07 | 2.11E-06 | *-* | hypothetical protein |
| MS14413_RS05190 | -2.08 | 1.06E-06 | *-* | fatty acid desaturase family protein |
| MS14413_RS16320 | -2.08 | 2.67E-10 | *-* | 3-oxoacyl-ACP reductase |
| MS14413_RS01605 | -2.09 | 2.38E-10 | *-* | O-methyltransferase |
| MS14413_RS04915 | -2.09 | 2.38E-10 | *-* | YecA family protein |
| MS14413_RS13470 | -2.10 | 1.23E-10 | *-* | thioesterase family protein |
| MS14413_RS02855 | -2.11 | 8.17E-08 | *-* | hypothetical protein |
| MS14413_RS10625 | -2.13 | 1.18E-08 | *-* | MerR family transcriptional regulator |
| MS14413_RS16215 | -2.14 | 5.12E-09 | *-* | N-acetyltransferase |
| MS14413_RS15345 | -2.15 | 1.08E-05 | *-* | hypothetical protein |
| MS14413_RS11455 | -2.15 | 2.29E-08 | *tatB* | twin-arginine translocase subunit TatB |
| MS14413_RS13240 | -2.15 | 3.10E-08 | *-* | pirin family protein |
| MS14413_RS18735 | -2.17 | 1.71E-06 | *-* | MBL fold metallo-hydrolase |
| MS14413_RS02705 | -2.19 | 2.28E-09 | *-* | septal ring lytic transglycosylase RlpA family protein |
| MS14413_RS01985 | -2.19 | 1.17E-10 | *crp* | cAMP-activated global transcriptional regulator CRP |
| MS14413_RS12360 | -2.20 | 1.06E-10 | *rpe* | ribulose-phosphate 3-epimerase |
| MS14413_RS11855 | -2.21 | 2.40E-10 | *-* | TetR/AcrR family transcriptional regulator |
| MS14413_RS17075 | -2.23 | 1.89E-08 | *-* | Lrp/AsnC family transcriptional regulator |
| MS14413_RS14440 | -2.26 | 1.13E-08 | *-* | esterase |
| MS14413_RS06730 | -2.27 | 2.18E-10 | *-* | acyl-CoA dehydrogenase C-terminal domain-containing protein |
| MS14413_RS02600 | -2.27 | 7.25E-09 | *-* | multidrug efflux SMR transporter AbeS |
| MS14413_RS10580 | -2.27 | 2.41E-11 | *-* | alpha/beta fold hydrolase |
| MS14413_RS15445 | -2.29 | 4.43E-11 | *-* | NADPH-dependent 2,2C4-dienoyl-CoA reductase |
| MS14413_RS09860 | -2.29 | 4.33E-09 | *-* | DMT family transporter |
| MS14413_RS10595 | -2.30 | 1.38E-09 | *-* | DUF1311 domain-containing protein |
| MS14413_RS05860 | -2.33 | 1.13E-09 | *-* | hypothetical protein |
| MS14413_RS06415 | -2.33 | 6.35E-08 | *-* | membrane protein |
| MS14413_RS04920 | -2.34 | 2.02E-10 | *-* | hypothetical protein |
| MS14413_RS17165 | -2.35 | 1.04E-08 | *-* | alpha/beta fold hydrolase |
| MS14413_RS10660 | -2.36 | 7.05E-09 | *-* | hypothetical protein |
| MS14413_RS02850 | -2.39 | 9.50E-06 | *-* | DUF2726 domain-containing protein |
| MS14413_RS14625 | -2.40 | 2.63E-09 | *-* | 5-formyltetrahydrofolate cyclo-ligase |
| MS14413_RS02890 | -2.41 | 2.02E-10 | *-* | tyrosine-type recombinase/integrase |
| MS14413_RS05140 | -2.42 | 2.76E-09 | *-* | MmcQ/YjbR family DNA-binding protein |
| MS14413_RS03475 | -2.42 | 1.65E-09 | *yddG* | aromatic amino acid DMT transporter YddG |
| MS14413_RS19105 | -2.42 | 1.39E-10 | *-* | acyl-CoA dehydrogenase family protein |
| MS14413_RS09900 | -2.43 | 1.20E-11 | *-* | DUF2236 domain-containing protein |
| MS14413_RS10665 | -2.45 | 1.79E-09 | *-* | peptidoglycan hydrolase |
| MS14413_RS09850 | -2.48 | 3.04E-08 | *-* | MFS transporter |
| MS14413_RS09855 | -2.49 | 1.07E-10 | *-* | TetR/AcrR family transcriptional regulator |
| MS14413_RS18510 | -2.50 | 5.15E-11 | *modA* | molybdate ABC transporter substrate-binding protein |
| MS14413_RS09845 | -2.50 | 1.07E-06 | *-* | helix-turn-helix domain-containing protein |
| MS14413_RS08765 | -2.53 | 4.68E-09 | *-* | matrixin family metalloprotease |
| MS14413_RS16285 | -2.53 | 2.60E-11 | *benE* | benzoate/H(+) symporter BenE family transporter |
| MS14413_RS16315 | -2.60 | 7.05E-11 | *-* | acetyl-CoA C-acetyltransferase |
| MS14413_RS14635 | -2.62 | 4.69E-08 | *-* | hypothetical protein |
| MS14413_RS17135 | -2.64 | 2.45E-08 | *-* | hypothetical protein |
| MS14413_RS02225 | -2.69 | 2.05E-10 | *-* | alpha/beta fold hydrolase |
| MS14413_RS18725 | -2.72 | 1.46E-07 | *-* | sulfite exporter TauE/SafE family protein |
| MS14413_RS18630 | -2.74 | 1.59E-08 | *-* | hypothetical protein |
| MS14413_RS11970 | -2.81 | 1.41E-11 | *-* | crotonase/enoyl-CoA hydratase family protein |
| MS14413_RS04220 | -2.92 | 2.41E-11 | *-* | enoyl-CoA hydratase/isomerase family protein |
| MS14413_RS16015 | -2.93 | 2.02E-10 | *-* | type II toxin-antitoxin system RelB/DinJ family antitoxin |
| MS14413_RS10970 | -3.02 | 3.47E-10 | *-* | hypothetical protein |
| MS14413_RS10975 | -3.08 | 2.41E-11 | *-* | tRNA-binding protein |
| MS14413_RS16740 | -3.22 | 1.56E-09 | *-* | Arc family DNA-binding protein |
| MS14413_RS11850 | -3.36 | 1.41E-11 | *-* | TetR/AcrR family transcriptional regulator |
| MS14413_RS18635 | -3.57 | 1.52E-09 | *-* | hypothetical protein |
| MS14413_RS16915 | -3.70 | 1.70E-10 | *-* | hypothetical protein |
| MS14413_RS00680 | -4.31 | 1.32E-12 | *-* | LysE family translocator |
| MS14413_RS15335 | -4.57 | 6.97E-13 | *-* | amino acid transporter |

**TABLE S2E** *A. baumannii* MS14413 up-regulated genes following 1 h treatment with 64 μM PBT2, 8 μM ZnSO_4_ and 1 μg/mL tetracycline in CA-MHB grown at 37°C.

| **Locus_tag** | **LogFC** | **FDR** | **Gene** | **Product** |
| --- | --- | --- | --- | --- |
| MS14413_RS02630 | 7.51 | 9.22E-10 | *adeF* | multidrug efflux RND transporter periplasmic adaptor subunit AdeF |
| MS14413_RS02635 | 6.26 | 9.03E-11 | *adeG* | multidrug efflux RND transporter permease subunit AdeG |
| MS14413_RS07940 | 5.99 | 1.01E-09 | *cntO* | TonB-dependent receptor |
| MS14413_RS19085 | 5.27 | 1.07E-09 | *-* | siderophore biosynthesis protein |
| MS14413_RS07060 | 5.02 | 8.79E-11 | *-* | (2Fe-2S)-binding protein |
| MS14413_RS19080 | 4.95 | 1.69E-09 | *-* | SidA/IucD/PvdA family monooxygenase |
| MS14413_RS02640 | 4.68 | 6.77E-10 | *adeH* | multidrug efflux RND transporter outer membrane subunit AdeH |
| MS14413_RS01755 | 4.45 | 1.80E-07 | *-* | transposase |
| MS14413_RS19075 | 4.39 | 4.44E-09 | *-* | DHA2 family efflux MFS transporter permease subunit |
| MS14413_RS15330 | 4.38 | 1.27E-12 | *czcE* | cation diffusion facilitator transporter |
| MS14413_RS15815 | 4.17 | 2.21E-10 | *-* | hypothetical protein |
| MS14413_RS15415 | 4.07 | 2.22E-09 | *cntO* | TonB-dependent receptor |
| MS14413_RS18965 | 3.97 | 3.67E-09 | *fpvA* | TonB-dependent siderophore receptor |
| MS14413_RS18485 | 3.81 | 1.50E-08 | *-* | transcriptional repressor |
| MS14413_RS00375 | 3.79 | 8.78E-09 | *arsC* | arsenate reductase (glutaredoxin) |
| MS14413_RS01730 | 3.69 | 2.79E-09 | *-* | cold-shock protein |
| MS14413_RS02750 | 3.68 | 2.50E-08 | *-* | OmpW family protein |
| MS14413_RS15810 | 3.62 | 2.45E-12 | *-* | LLM class flavin-dependent oxidoreductase |
| MS14413_RS18015 | 3.58 | 3.60E-10 | *-* | MFS transporter |
| MS14413_RS14360 | 3.52 | 1.43E-09 | *pfeA* | TonB-dependent siderophore receptor |
| MS14413_RS04320 | 3.45 | 1.98E-09 | *-* | CSLREA domain-containing protein |
| MS14413_RS16225 | 3.34 | 1.23E-07 | *-* | TonB-dependent siderophore receptor |
| MS14413_RS02495 | 3.34 | 3.09E-11 | *-* | alpha/beta hydrolase |
| MS14413_RS03395 | 3.25 | 1.96E-06 | *bauD* | ferric acinetobactin ABC transporter permease subunit BauD |
| MS14413_RS18310 | 3.21 | 3.59E-09 | *adeA* | multidrug efflux RND transporter periplasmic adaptor subunit AdeA |
| MS14413_RS11500 | 3.19 | 7.15E-10 | *-* | TonB-dependent siderophore receptor |
| MS14413_RS10340 | 3.11 | 3.31E-11 | *feoA* | ferrous iron transport protein A |
| MS14413_RS06785 | 3.09 | 1.58E-08 | *-* | EamA family transporter |
| MS14413_RS05345 | 3.07 | 3.31E-11 | *-* | PepSY domain-containing protein |
| MS14413_RS01225 | 3.06 | 4.50E-07 | *-* | hypothetical protein |
| MS14413_RS00365 | 3.00 | 1.04E-07 | *arsB* | ACR3 family arsenite efflux transporter |
| MS14413_RS03390 | 2.98 | 2.63E-06 | *bauC* | ferric acinetobactin ABC transporter permease subunit BauC |
| MS14413_RS18970 | 2.92 | 1.42E-06 | *-* | hypothetical protein |
| MS14413_RS03410 | 2.92 | 2.05E-09 | *bauF* | acinetobactin utilization protein BauF |
| MS14413_RS18650 | 2.92 | 3.33E-09 | *-* | MFS transporter |
| MS14413_RS10195 | 2.90 | 1.61E-09 | *-* | IS*91*-like element IS*Vsa3* family transposase |
| MS14413_RS03400 | 2.89 | 2.54E-07 | *basB* | acinetobactin non-ribosomal peptide synthetase subunit BasB |
| MS14413_RS17305 | 2.88 | 1.01E-09 | *-* | TonB-dependent siderophore receptor |
| MS14413_RS07085 | 2.86 | 5.29E-07 | *-* | pilin |
| MS14413_RS19070 | 2.85 | 5.56E-09 | *acsC* | siderophore achromobactin biosynthesis protein AcsC |
| MS14413_RS03355 | 2.85 | 5.35E-08 | *basF* | acinetobactin biosynthesis bifunctional isochorismatase/aryl carrier protein BasF |
| MS14413_RS07500 | 2.84 | 4.79E-08 | *-* | helix-turn-helix transcriptional regulator |
| MS14413_RS18975 | 2.83 | 1.98E-07 | *-* | PepSY domain-containing protein |
| MS14413_RS00765 | 2.74 | 2.18E-07 | *-* | hypothetical protein |
| MS14413_RS03360 | 2.73 | 1.40E-07 | *basE* | (2,2C3-dihydroxybenzoyl)adenylate synthase BasE |
| MS14413_RS19050 | 2.73 | 6.92E-09 | *-* | TonB-dependent receptor |
| MS14413_RS19065 | 2.69 | 5.73E-09 | *-* | IucA/IucC family siderophore biosynthesis protein |
| MS14413_RS01725 | 2.69 | 5.30E-07 | *-* | hypothetical protein |
| MS14413_RS00640 | 2.64 | 8.55E-07 | *-* | amino acid ABC transporter permease |
| MS14413_RS00645 | 2.62 | 2.00E-07 | *-* | amino acid ABC transporter permease |
| MS14413_RS07625 | 2.62 | 2.04E-07 | *-* | AzlD domain-containing protein |
| MS14413_RS19045 | 2.59 | 3.82E-07 | *-* | hypothetical protein |
| MS14413_RS10200 | 2.56 | 1.55E-10 | *-* | hypothetical protein |
| MS14413_RS04145 | 2.55 | 4.83E-08 | *-* | sulfate ABC transporter substrate-binding protein |
| MS14413_RS08245 | 2.54 | 9.11E-08 | *-* | amino acid permease |
| MS14413_RS17450 | 2.53 | 9.29E-08 | *-* | 3-oxoacid CoA-transferase subunit A |
| MS14413_RS17850 | 2.53 | 2.51E-08 | *-* | TetR/AcrR family transcriptional regulator |
| MS14413_RS13485 | 2.52 | 1.07E-08 | *ggt* | gamma-glutamyltransferase |
| MS14413_RS11385 | 2.52 | 1.04E-08 | *-* | energy transducer TonB |
| MS14413_RS19265 | 2.52 | 1.46E-08 | *-* | ABC transporter substrate-binding protein |
| MS14413_RS06690 | 2.51 | 2.58E-07 | *-* | DEAD/DEAH box helicase |
| MS14413_RS07665 | 2.51 | 6.13E-09 | *-* | TetR/AcrR family transcriptional regulator |
| MS14413_RS11120 | 2.47 | 7.46E-10 | *-* | Rne/Rng family ribonuclease |
| MS14413_RS03385 | 2.46 | 1.76E-06 | *bauE* | ferric acinetobactin ABC transporter ATP-binding protein BauE |
| MS14413_RS18495 | 2.44 | 4.95E-09 | *-* | SDR family oxidoreductase |
| MS14413_RS12450 | 2.42 | 8.30E-07 | *-* | MFS transporter |
| MS14413_RS12580 | 2.41 | 1.67E-06 | *-* | hypothetical protein |
| MS14413_RS10190 | 2.41 | 2.62E-07 | *-* | hypothetical protein |
| MS14413_RS07470 | 2.40 | 4.85E-09 | *-* | hypothetical protein |
| MS14413_RS18190 | 2.40 | 1.29E-08 | *iaaH* | indoleacetamide hydrolase |
| MS14413_RS18010 | 2.40 | 6.33E-09 | *-* | HlyD family secretion protein |
| MS14413_RS00430 | 2.37 | 4.35E-08 | *tauA* | taurine ABC transporter substrate-binding protein |
| MS14413_RS08250 | 2.36 | 9.26E-08 | *hutH* | histidine ammonia-lyase |
| MS14413_RS06970 | 2.36 | 5.11E-07 | *-* | triacylglycerol lipase |
| MS14413_RS00635 | 2.35 | 6.17E-06 | *-* | amino acid ABC transporter ATP-binding protein |
| MS14413_RS00370 | 2.34 | 3.37E-07 | *-* | metalloregulator ArsR/SmtB family transcription factor |
| MS14413_RS17475 | 2.34 | 1.37E-06 | *-* | MFS transporter |
| MS14413_RS14185 | 2.34 | 2.60E-07 | *-* | non-heme iron oxygenase ferredoxin subunit |
| MS14413_RS18215 | 2.34 | 2.18E-07 | *-* | TolC family protein |
| MS14413_RS02425 | 2.32 | 1.68E-09 | *-* | cold-shock protein |
| MS14413_RS07860 | 2.32 | 3.31E-11 | *-* | TonB-dependent siderophore receptor |
| MS14413_RS16185 | 2.31 | 3.77E-09 | *-* | TonB-dependent receptor |
| MS14413_RS01625 | 2.29 | 3.88E-08 | *-* | HlyD family type I secretion periplasmic adaptor subunit |
| MS14413_RS00140 | 2.29 | 3.49E-08 | *-* | hypothetical protein |
| MS14413_RS19260 | 2.29 | 1.02E-08 | *-* | Zn-dependent oligopeptidase |
| MS14413_RS06965 | 2.28 | 4.71E-07 | *-* | lipase secretion chaperone |
| MS14413_RS09975 | 2.28 | 3.73E-09 | *-* | DUF2726 domain-containing protein |
| MS14413_RS18200 | 2.27 | 4.17E-06 | *-* | DHA2 family efflux MFS transporter permease subunit |
| MS14413_RS00790 | 2.27 | 4.08E-08 | *-* | methylcrotonoyl-CoA carboxylase |
| MS14413_RS01150 | 2.27 | 2.93E-09 | *-* | L-serine ammonia-lyase |
| MS14413_RS17845 | 2.26 | 1.84E-07 | *-* | MBL fold metallo-hydrolase |
| MS14413_RS01630 | 2.26 | 4.21E-08 | *-* | type I secretion system permease/ATPase |
| MS14413_RS04645 | 2.25 | 1.61E-09 | *-* | Ig-like domain repeat protein |
| MS14413_RS18435 | 2.24 | 9.11E-08 | *-* | hypothetical protein |
| MS14413_RS00115 | 2.23 | 1.32E-06 | *-* | cytosine permease |
| MS14413_RS06860 | 2.23 | 8.75E-10 | *-* | hypothetical protein |
| MS14413_RS18980 | 2.23 | 2.09E-06 | *-* | hypothetical protein |
| MS14413_RS03365 | 2.22 | 2.03E-07 | *basD* | acinetobactin non-ribosomal peptide synthetase subunit BasD |
| MS14413_RS17470 | 2.22 | 1.07E-06 | *pcaD* | 3-oxoadipate enol-lactonase |
| MS14413_RS17940 | 2.20 | 1.02E-06 | *-* | SLC13 family permease |
| MS14413_RS08595 | 2.20 | 1.83E-07 | *-* | hypothetical protein |
| MS14413_RS07750 | 2.20 | 5.73E-09 | *-* | hypothetical protein |
| MS14413_RS10345 | 2.19 | 9.33E-10 | *feoB* | ferrous iron transporter B |
| MS14413_RS09570 | 2.19 | 1.11E-08 | *-* | AMP-binding protein |
| MS14413_RS00345 | 2.18 | 6.33E-09 | *ahpF* | alkyl hydroperoxide reductase subunit F |
| MS14413_RS00515 | 2.16 | 3.91E-06 | *-* | malonate decarboxylase subunit delta |
| MS14413_RS16110 | 2.15 | 1.17E-05 | *-* | iron-containing alcohol dehydrogenase |
| MS14413_RS03350 | 2.15 | 6.50E-07 | *basG* | acinetobactin biosynthesis histidine decarboxylase BasG |
| MS14413_RS08905 | 2.14 | 4.23E-06 | *-* | sulfonate ABC transporter substrate-binding protein |
| MS14413_RS17465 | 2.14 | 1.85E-07 | *-* | 3-carboxy-cis,2Ccis-muconate cycloisomerase |
| MS14413_RS09565 | 2.13 | 1.74E-08 | *mmsB* | 3-hydroxyisobutyrate dehydrogenase |
| MS14413_RS03775 | 2.12 | 3.14E-09 | *-* | ferredoxin reductase |
| MS14413_RS07620 | 2.12 | 2.33E-07 | *-* | AzlC family ABC transporter permease |
| MS14413_RS02880 | 2.11 | 3.82E-07 | *-* | peptide MFS transporter |
| MS14413_RS18315 | 2.11 | 3.06E-08 | *adeB* | multidrug efflux RND transporter permease subunit AdeB |
| MS14413_RS15965 | 2.11 | 1.56E-05 | *-* | hypothetical protein |
| MS14413_RS09550 | 2.09 | 2.73E-09 | *-* | amino acid permease |
| MS14413_RS19035 | 2.08 | 1.79E-05 | *-* | hypothetical protein |
| MS14413_RS10205 | 2.07 | 8.02E-10 | *-* | aminoglycoside O-phosphotransferase APH(6)-Id |
| MS14413_RS18195 | 2.07 | 7.75E-06 | *-* | HlyD family secretion protein |
| MS14413_RS12500 | 2.06 | 2.48E-07 | *-* | AraC family transcriptional regulator |
| MS14413_RS01890 | 2.05 | 6.77E-10 | *ahpF* | alkyl hydroperoxide reductase subunit F |
| MS14413_RS00795 | 2.03 | 3.92E-07 | *-* | enoyl-CoA hydratase/isomerase family protein |
| MS14413_RS02540 | 2.03 | 1.24E-08 | *-* | hemin uptake protein HemP |
| MS14413_RS06360 | 2.03 | 1.90E-06 | *-* | HAD-IB family hydrolase |
| MS14413_RS01155 | 2.03 | 1.97E-08 | *-* | APC family permease |
| MS14413_RS00785 | 2.03 | 5.18E-09 | *-* | isovaleryl-CoA dehydrogenase |
| MS14413_RS18785 | 2.02 | 8.28E-08 | *-* | transcriptional regulator |
| MS14413_RS12310 | 2.02 | 1.41E-07 | *filE* | putative pilus assembly protein FilE |
| MS14413_RS01720 | 2.02 | 1.37E-06 | *-* | NAD(P)-binding domain-containing protein |

**TABLE S2F** *A. baumannii* MS14413 down-regulated genes following 1 h treatment with 64 μM PBT2, 8 μM ZnSO_4_ and 1 μg/mL tetracycline in CA-MHB grown at 37°C.

| **Locus_tag** | **LogFC** | **FDR** | **Gene** | **Product** |
| --- | --- | --- | --- | --- |
| MS14413_RS16490 | -2.01 | 3.34E-3 | *-* | hypothetical protein |
| MS14413_RS12210 | -2.01 | 1.72E-10 | *gigB* | anti-anti-sigma factor GigB |
| MS14413_RS11855 | -2.03 | 5.81E-10 | *-* | TetR/AcrR family transcriptional regulator |
| MS14413_RS11455 | -2.05 | 3.49E-08 | *tatB* | twin-arginine translocase subunit TatB |
| MS14413_RS12360 | -2.08 | 1.63E-10 | *rpe* | ribulose-phosphate 3-epimerase |
| MS14413_RS17390 | -2.10 | 1.69E-09 | *-* | NADPH-dependent 2,2C4-dienoyl-CoA reductase |
| MS14413_RS13470 | -2.10 | 1.37E-10 | *-* | thioesterase family protein |
| MS14413_RS18630 | -2.11 | 1.08E-07 | *-* | hypothetical protein |
| MS14413_RS14635 | -2.11 | 2.29E-07 | *-* | hypothetical protein |
| MS14413_RS06730 | -2.14 | 4.27E-10 | *-* | acyl-CoA dehydrogenase C-terminal domain-containing protein |
| MS14413_RS14440 | -2.17 | 1.50E-08 | *-* | esterase |
| MS14413_RS10580 | -2.18 | 3.31E-11 | *-* | alpha/beta fold hydrolase |
| MS14413_RS17075 | -2.19 | 2.37E-08 | *-* | Lrp/AsnC family transcriptional regulator |
| MS14413_RS04920 | -2.20 | 3.72E-10 | *-* | hypothetical protein |
| MS14413_RS09845 | -2.21 | 3.20E-06 | *-* | helix-turn-helix domain-containing protein |
| MS14413_RS08765 | -2.21 | 1.32E-08 | *-* | matrixin family metalloprotease |
| MS14413_RS04960 | -2.21 | 7.22E-08 | *-* | helix-turn-helix transcriptional regulator |
| MS14413_RS02890 | -2.22 | 4.39E-10 | *-* | tyrosine-type recombinase/integrase |
| MS14413_RS04915 | -2.22 | 1.93E-10 | *-* | YecA family protein |
| MS14413_RS05805 | -2.23 | 4.05E-10 | *-* | acyl-CoA dehydrogenase C-terminal domain-containing protein |
| MS14413_RS17135 | -2.25 | 7.98E-08 | *-* | hypothetical protein |
| MS14413_RS14625 | -2.29 | 4.24E-09 | *-* | 5-formyltetrahydrofolate cyclo-ligase |
| MS14413_RS18725 | -2.30 | 4.95E-07 | *-* | sulfite exporter TauE/SafE family protein |
| MS14413_RS09860 | -2.32 | 4.34E-09 | *-* | DMT family transporter |
| MS14413_RS01435 | -2.33 | 9.74E-07 | *-* | hypothetical protein |
| MS14413_RS19105 | -2.33 | 2.06E-10 | *-* | acyl-CoA dehydrogenase family protein |
| MS14413_RS09900 | -2.34 | 1.40E-11 | *-* | DUF2236 domain-containing protein |
| MS14413_RS05140 | -2.35 | 4.07E-09 | *-* | MmcQ/YjbR family DNA-binding protein |
| MS14413_RS05800 | -2.36 | 6.47E-10 | *-* | acyl-CoA dehydrogenase C-terminal domain-containing protein |
| MS14413_RS15445 | -2.38 | 3.31E-11 | *-* | NADPH-dependent 2,2C4-dienoyl-CoA reductase |
| MS14413_RS16015 | -2.38 | 9.25E-10 | *-* | type II toxin-antitoxin system RelB/DinJ family antitoxin |
| MS14413_RS17165 | -2.40 | 8.62E-09 | *-* | alpha/beta fold hydrolase |
| MS14413_RS02600 | -2.44 | 4.93E-09 | *abeS* | multidrug efflux SMR transporter AbeS |
| MS14413_RS02225 | -2.45 | 4.69E-10 | *-* | alpha/beta fold hydrolase |
| MS14413_RS18730 | -2.46 | 4.66E-07 | *-* | TIGR01244 family phosphatase |
| MS14413_RS16315 | -2.47 | 1.13E-10 | *-* | acetyl-CoA C-acetyltransferase |
| MS14413_RS09850 | -2.50 | 2.80E-08 | *-* | MFS transporter |
| MS14413_RS03475 | -2.51 | 1.61E-09 | *yddG* | aromatic amino acid DMT transporter YddG |
| MS14413_RS09855 | -2.54 | 9.03E-11 | *-* | TetR/AcrR family transcriptional regulator |
| MS14413_RS16285 | -2.54 | 3.13E-11 | *benE* | benzoate/H(+) symporter BenE family transporter |
| MS14413_RS18510 | -2.60 | 3.31E-11 | *modA* | molybdate ABC transporter substrate-binding protein |
| MS14413_RS18735 | -2.62 | 3.67E-07 | *blh* | MBL fold metallo-hydrolase |
| MS14413_RS16740 | -2.70 | 4.77E-09 | *-* | Arc family DNA-binding protein |
| MS14413_RS18635 | -2.82 | 5.49E-09 | *-* | hypothetical protein |
| MS14413_RS04220 | -2.98 | 2.12E-11 | *-* | enoyl-CoA hydratase/isomerase family protein |
| MS14413_RS11970 | -3.06 | 7.93E-12 | *-* | crotonase/enoyl-CoA hydratase family protein |
| MS14413_RS16915 | -3.08 | 4.69E-10 | *-* | hypothetical protein |
| MS14413_RS10970 | -3.17 | 3.02E-10 | *-* | hypothetical protein |
| MS14413_RS10975 | -3.22 | 1.97E-11 | *-* | tRNA-binding protein |
| MS14413_RS11850 | -3.42 | 1.27E-11 | *-* | TetR/AcrR family transcriptional regulator |
| MS14413_RS00680 | -4.35 | 1.71E-12 | *-* | LysE family translocator |
| MS14413_RS15335 | -4.74 | 6.31E-13 | *-* | amino acid transporter |
