# Supplementary material for: Rescuing Tetracycline Class Antibiotics for the Treatment of Multidrug-Resistant Acinetobacter baumannii Pulmonary Infection
Source: mBio. 2022 Jan 11;13(1):e03517-21. doi: 10.1128/mbio.03517-21 (PMC8749419; doi:10.1128/mbio.03517-21)
Supplement: TABLE S3 [file mbio.03517-21-st003.docx]

**TABLE S3** Primers used in this study.

| ***A. baumannii* MS14413** | | |
| --- | --- | --- |
| *PCR primers* | | |
| Gene | Primer direction | Sequence 5’🡪3’ |
| *adeN* | Fwd | ATGCATGATCCAGTCCTTGAG |
| *adeN* | Rev | TTAGACTTTATGATGGCCCTTT |
| *adeS* | Fwd | ATGAAAAGTAAGTTAGGAATT |
| *adeS* | Rev | TTAGTTATTCATAGAAATTTT |
| *Quantitative real-time PCR primers* | | |
| Gene | Primer direction | Sequence 5’🡪3’ |
| *adeA* | Fwd | GTCAGGCTCTAGCCGATGTC |
| *adeA* | Rev | ATGGTTGCCATCGTATTGGT |
| *adeB* | Fwd | CATGTTCGGTATGGTGCTTG |
| *adeB* | Rev | AATACTGCCGCCAATACCAG |
| *adeC* | Fwd | GTCATCCTGTTCCGCAAAAT |
| *adeC* | Rev | TTGCTCCTGCAACACGTAAC |
| *adeF* | Fwd | CCGTGGGTAATGTCGTCTCT |
| *adeF* | Rev | CGGATCGTACCTGAGGTTGT |
| *adeG* | Fwd | TTGCTGCACAAACTCTACCG |
| *adeG* | Rev | CTCCAGCTGTCAACCAGACA |
| *adeH* | Fwd | TCAGGCTTCACGTGCATTAC |
| *adeH* | Rev | TTGCTGTAGATCCGCTGTTG |
| *feoA* | Rev | CAGCGTTGAAAGTGAAGCAA |
| *feoA* | Fwd | GGATCACCACCAAAAACACC |
| *feoB* | Fwd | GCAGGTGGCGTAGTTGTTTT |
| *feoB* | Rev | AATTCCCGGTACTGCACAAG |
| *bauC* | Rev | ACATCCAAAACAGGGAGTGC |
| *bauC* | Fwd | CGTTGGGACGGTTACTCAAT |
| *bauD* | Fwd | GCCACCCTTGCATACTTTGT |
| *bauD* | Rev | GCACGCTGAGCAAGATGATA |
| *bauE* | Fwd | CAGCCACGCTCACTGTATGT |
| *bauE* | Rev | CTCAGGCGCTTATGTGATGA |
| *abeS* | Rev | ACGGTTGTGGGTTATGCAGT |
| *abeS* | Fwd | AACCAATACAGGCAGCCAAG |
| *recA* | Fwd | GGCCAATTTTATTGCCTTGA |
| *recA* | Rev | CAAAATGGCTCCTCCGTTTA |
